# Synthesis of single-crystal-like nanoporous carbon membranes and their application in overall water splitting

**DOI:** 10.1038/ncomms13592

**Published:** 2017-01-04

**Authors:** Hong Wang, Shixiong Min, Chun Ma, Zhixiong Liu, Weiyi Zhang, Qiang Wang, Debao Li, Yangyang Li, Stuart Turner, Yu Han, Haibo Zhu, Edy Abou-hamad, Mohamed Nejib Hedhili, Jun Pan, Weili Yu, Kuo-Wei Huang, Lain-Jong Li, Jiayin Yuan, Markus Antonietti, Tom Wu

**Affiliations:** 1 Physical Science and Engineering Division, King Abdullah University of Science & Technology, Thuwal 23955-6900, Saudi Arabia; 2 Kaust Catalysis Center, King Abdullah University of Science & Technology, Thuwal 23955-6900, Saudi Arabia; 3 Max Planck Institute of Colloids and Interfaces, Department of Colloid Chemistry, D-14476 Potsdam, Germany; 4 State Key Laboratory of Coal Conversion, Institute of Coal Chemistry, The Chinese Academy of Sciences, Taiyuan 030001, China; 5 EMAT, University of Antwerp, Groenenborgerlaan 171, B-2020 Antwerpen, Belgium; 6 Membrane Research Centre, King Abdullah University of Science and Technology, Thuwal 23955-6900, Saudi Arabia; 7 Imaging and Characterization Core Lab, King Abdullah University of Science and Technology, Thuwal 23955-6900, Saudi Arabia

## Abstract

Nanoporous graphitic carbon membranes with defined chemical composition and pore architecture are novel nanomaterials that are actively pursued. Compared with easy-to-make porous carbon powders that dominate the porous carbon research and applications in energy generation/conversion and environmental remediation, porous carbon membranes are synthetically more challenging though rather appealing from an application perspective due to their structural integrity, interconnectivity and purity. Here we report a simple bottom–up approach to fabricate large-size, freestanding and porous carbon membranes that feature an unusual single-crystal-like graphitic order and hierarchical pore architecture plus favourable nitrogen doping. When loaded with cobalt nanoparticles, such carbon membranes serve as high-performance carbon-based non-noble metal electrocatalyst for overall water splitting.

## Abstract (web summary)

Hierarchical, nanoporous graphitic membranes are synthetically challenging yet attractive materials, due to their structural integrity. Here, the authors fabricate freestanding, porous carbon membranes with single-crystal-like graphitic order which serve as water splitting electrocatalysts upon cobalt doping.

Carbon materials have been widely used to address global energy and environmental issues due to their extraordinary, tuneable physicochemical properties, rich abundance and low cost^1^,^2^,^3^. Freestanding porous carbon membranes particularly hold great promise in the fields of catalysis, water treatment, biofiltration, gas separation and optoelectronics, just to name a few, due to their structural integrity, continuity and purity^4^,^5^. Typical synthetic methods involve mechanical rolling of thermally expanded graphite flakes, chemical vapour deposition and vacuum filtration of dispersions of graphene sheets or carbon nanotubes^6^,^7^,^8^,^9^,^10^. In addition, Koros and co-works^11^,^12^,^13^ reported that pyrolysis of thermosetting polymer precursors (for example, aromatic polyimides) could lead to carbon membrane sieves with micropores, which exhibited high-performance for gas separation. For some carbon-based energy applications, such as electrodes in electrochemical energy conversion/storage, and nanoelectronic devices, however, precise control over the atomic order, local chemical composition, nanoscale morphology and complex pore architecture, as well as easy access to porous membranes of large size and large surface area, is highly relevant but cannot be fully met by the state-of-the-art synthetic protocols. Particularly, a high degree of graphitization and hierarchical pore architecture with interconnected pores over a broad length scale are eagerly being pursued because they could offer fast electron conduction, and rapid mass transport through large pores along with a simultaneously high-reaction capacity via the large accessible surface area provided by the micro/mesopores^14^. Furthermore, the pores in the cross sections of the carbon membrane if distributed in a gradient manner, can offer unconventional fluidic transport on the nanoscale (for example, concentration gradient^15^ and permselectivity^16^,^17^ for broad application in micro/nano-fluidic devices^18^,^19^).

In this study, we report a bottom–up approach for fabrication of hierarchically structured, nitrogen-doped, graphitic nanoporous carbon membranes (termed HNDCMs) via morphology retaining carbonization of a porous polymer membrane precursor. In particular, the pores along the membrane cross-section assume a gradient distribution in their sizes, and the pore walls exhibit unusual single-crystal-like characteristics. As a prototypical application, when loaded with cobalt nanoparticles, these highly conductive porous carbon membranes serve as an active carbon-based bifunctional electrocatalyst for overall water splitting.

## Results

### Synthesis and structure characterization

**Figure 1:**
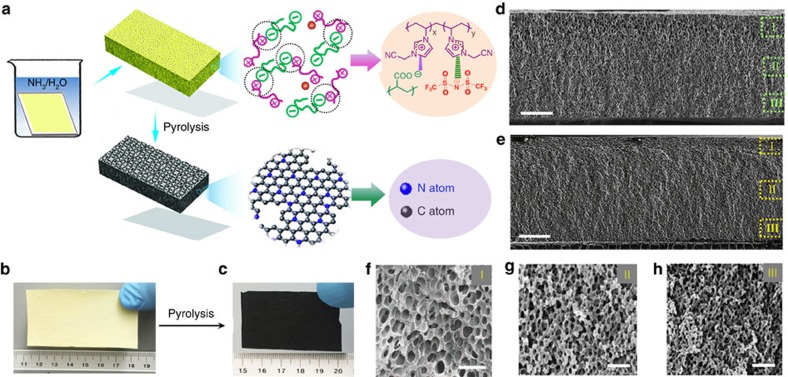
Formation and structure of hierarchically structured nitrogen-doped porous carbon membranes. (**a**) Schematic illustration of the preparation procedure. (**b**) Photograph of a 7.2 × 3.3 cm^2^ free-standing GPPM. (**c**) Photograph of a 5.2 × 2.5 cm^2^ free-standing HNDCM obtained by pyrolysis of GPPM in **b,** the unit in the ruler in **b** and **c** is centimetre. (**d**) Cross-section scanning electron microscopy (SEM) image of the HNDCM-100,000-1,000. (**e**) SEM image of the cross section of HNDCM-250,000-1,000, the scale bars in **d**,**e** are 20 μm. (**f**–**h**) High-magnification SEM images of the cross-section structures of HNDCM-250,000–1,000. The scale bars represent 500 nm.

Figure 1a shows the membrane fabrication process. First, porous polyelectrolyte membranes bearing gradually varying pore sizes along the membrane cross section (termed GPPMs, gradient porous polymer membranes) were assembled according to a previously reported procedure by exploiting electrostatic cross-linking, that is, interpolyelectrolyte complexation between cationic poly[1-cyanomethyl-3-vinylimidazolium bis(trifluoromethanesulfonyl)imide] (PCMVImTf_2_N) and anionic neutralized poly(acrylic acid) (PAA)^20^. The structural characterization of PCMVImTf_2_N (Supplementary Figs 1–3) and details of the polymer membrane fabrication method are provided in the supporting materials. It is important to note that the preparation of GPPMs is a mature and robust technique that can produce various pore profiles at desirable size scales. Next, direct pyrolysis of the free-standing GPPMs under a nitrogen flow yielded HNDCMs. For example, carbonization of a rectangular GPPM that was 7.2 × 3.3 cm^2^ in size and 96 μm in thickness (Fig. 1b) produced a HNDCM that was 5.2 × 2.5 cm^2^ in size and 62 μm thick (Fig. 1c). Shrinkage of the membrane dimensions during pyrolysis was accompanied by a weight reduction of 75%.

Importantly, the pore architectures of the carbon membranes can be regulated by the molecular weight (MW) of the polymeric precursors. This correlation was investigated by pairing the same PCMVImTf_2_N with PAAs of different MW. Here, the GPPM-x and HNDCM-x-y notations are used, where *x* and *y* denote the MW of PAA and the carbonization temperature, respectively. These two crucial parameters are carefully paired to prepare carbon membranes with the desirable characteristics. For example, GPPM-2000 exhibited an interconnected porous network but its carbon product at 1,000 °C (that is, HNDCM-2000–1000) only possessed inconsecutive pores (Supplementary Fig. 4). In fact, the interconnected pores in GPPM-2000 were blocked even at 300 °C (Supplementary Fig. 5). Surprisingly, pyrolysis of GPPM-100,000 (Supplementary Fig. 6) at 1,000 °C preserves the well-defined porous morphology (Fig. 1d), and an asymmetric, three-dimensionally interconnected pore architecture was spontaneously created in HNDCM-100,000–1,000. From the top to the bottom, the pore size of HNDCM-100,000-1,000 gradually decreased from 1.5 μm and 900–550 nm in zones I, II and III. Impressively, in sample HNDCM-250,000-1,000, the pore sizes (Fig. 1e–h) decreased to 250±10 nm, 75±8 nm and 32±6 nm in zones I, II and III, respectively, indicating that the pore size can be readily tuned by the MW of PAA. As observed, the pore morphology of the carbon membrane is position-specific, that is, from larger ones on the top gradually to smaller ones at the bottom. It is actually a natural outcome of the cross-linking density profile in the GPPM template. The higher the cross-linking density in the polymer membrane is, the larger the pores in the carbon membrane will be, because dense cross-linking undermines the trimerization reaction of cyano groups that require sufficient mobility to reach desirable positions to complete the triazine ring formation. Therefore, the spatial restriction promotes thermodegradation of the porous polymer network in the highly cross-linked top region, which is further amplified by larger pores in the same area, forming carbon pores with larger size than those at the bottom. For PAA with an even higher MW of 450,000 and 3,000,000, the carbon membranes were highly porous but became too fragile on carbonization (Supplementary Figs 7 and 8). In general, pyrolysis enlarges the pore size in HNDCMs compared with that in GPPMs due to the considerable mass loss in the form of volatile species during carbonization (Supplementary Fig. 9).

### Formation mechanism

The relationship between the porous morphology and the MW of PAA provides a practical route to tailor the membrane structure, which is a natural outcome of the function of PAA in the synthesis of GPPMs. PAA acts as a crosslinker to chemically lock PCMVImTf_2_N in a porous network via electrostatic complexation. In addition, the cross-linking density in the GPPMs increased as the MW of PAA increased (Supplementary Table 1). The collapse of GPPM-2000 at temperatures above 300 °C was due to the relatively low cross-linking density (that is, pores too large), which cannot stabilize the pores; the cracking of the carbon membranes prepared from PAA with a MW of ∼450,000 and 3,000,000 resulted from a cross-linking density that was too high (that is, pores too small), which results in the build-up of excessively high inner stress during the carbonization process. Only pyrolysis of polymer membranes with moderate MW ends up with retention of the structural integrity. According to the thermogravimetric analysis (Supplementary Fig. 10), these polymer membranes start to detach their alkyl chains and H and O elements at 280 °C, and a thermally induced trimerization reaction of the cyano groups simultaneously occurs to build up a stable triazine network^21^. It is important to note that in general, morphology retaining carbonization *via* pyrolysis of porous polymer precursors is challenging because pyrolysis typically breaks down polymeric chains and results in cracks^22^. Dai *et al*.^23^ reported that the cross-linking state of polymer precursors is a key to achieving crack-free carbon membranes. Here, our synthesis of HNDCMs demonstrates that the porous nano/microstructure in the carbon precursor can be preserved due to a synergistic combination of the initial crosslinked state of the precursors and the subsequent formation of a thermally stable network intermediate during the bottom–up carbonization process.

### Unique graphitic structures

**Figure 2:**
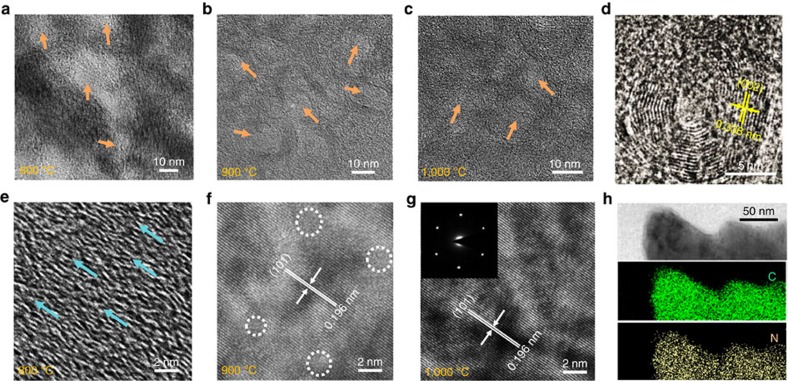
Microstructural characterizations of the N-doped porous carbon membrane. HRTEM images of (**a**,**e**) HNDCM-100,000-800, (**b**,**f**) HNDCM-100,000-900, (**c**,**g**) HNDCM-100,000-1,000 and (**d**) a typical onion-like graphitic structure in HNDCM-100,000-1,000. Some defective regions are highlighted in **f**. Inset in **g** is the selected-area electron diffraction (SAED) pattern for HNDCM-100,000-1,000. The SAED pattern indicates the single-crystal-like characteristics of HNDCM-100,000-1,000. (**h**) TEM image and corresponding elemental (C and N) mappings, scale bar, 50 nm.

High-resolution transmission electron microscopy (HRTEM) images provide insight into the microscopic and atomic structures of the HNDCM-100,000-y samples (*y*=800, 900 and 1,000) that were prepared using three different pyrolysis temperatures. Figure 2a shows the presence of mesopores that are 2–50 nm in size for HNDCM-100,000–800. Interestingly, as shown in Fig. 2b–d, we observed onion-like concentric graphitic nanostructures that consist of multi-shells and hollow cage-like centres. The shells are composed of (002) graphitic planes with a lattice spacing of 0.338±0.02 nm. The HRTEM images of HNDCM-100,000–800 displayed in Fig. 2e and Supplementary Fig. 11 show the preferential orientation of the graphitic layers. Unexpectedly, in HNDCM-100,000–900, a single-crystal-like atomic packing emerged in the entire membrane. The fringes show a well-defined lattice spacing of 0.196±0.02 nm (Fig. 2f and Supplementary Fig.12), which corresponds to the (101) plane of graphite^24^. Notably, HNDCM-100,000-1,000 has the same graphitic structure but with fewer lattice defects (Fig. 2g and Supplementary Fig. 13). A selected-area electron diffraction measurement (inset in Fig. 2g) yielded a sixfold symmetric spot pattern, which is characteristic of well-crystalized graphite. These results indicate progressive graphitization at elevated temperatures from 800 to 1,000 °C. A similar trend was confirmed by Raman, X-ray diffraction (XRD) and solid-state ^13^C-nuclear magnetic resonance (NMR) measurements (Supplementary Figs 14–16). Energy-filtered transmission electron microscopy mappings for both C and N (Fig. 2h) indicate a uniform distribution of N in the carbon matrix, which is expected due to *in situ* molecular doping of HNDCM with N. The synergy between the N lone pair and the *π*-system of the C lattice can dramatically alter the physicochemical properties of the HNDCMs (for example, oxidative stability and catalytic activity)^25^. For example, the HNDCM-100,000-1,000 sample is fire-retardant (Supplementary Fig. 17). Even in an acetylene flame (>1,000 °C) in air for 60 s, this sample maintained its original colour and morphology, which is indicative of its excellent oxidative stability and its potential for use as a fire-retardant protective material.

The elemental analysis indicated that the N content in HNDCM-100,000–800/900/1,000 membranes was 11.7 wt.%, 8.27 wt. % and 5.7 wt.%, respectively, which is in good accordance with the results from the X-ray photoelectron spectroscopy (XPS) analysis. As previously reported, a high N content may hinder the crystallinity of carbon^26^, which is in good agreement with our observation that despite a relatively lower N content, HNDCM-100,000-1,000 is more graphitic than the other two samples. Nevertheless, the high crystallinity of the membranes prepared at 900 and 1,000 °C is surprising because the formation of graphitic carbon from polymer precursors typically requires much higher pyrolysis temperatures. Previous studies have demonstrated that pyrolysis of carbon precursors above 800 °C in the presence of metal catalysts can improve graphitization^27^. However, HNDCMs are free of metal catalyst, as extra confirmed by XPS measurements (Supplementary Fig. 18 and Supplementary Table 3). Single-crystal-like carbons cannot be obtained by carbonization at 1,000 °C of either native PCMVImTf_2_N or its physical blend with PAA (Supplementary Fig. 19) as well as other polymeric precursors, such as polyacrylonitrile^28^ and poly(acrylamide-*co*-acrylic acid)^29^. It is important to note that PCMVImTf_2_N rather than PAA is the main carbon precursor for HNDCMs due to its high carbonization yield and being 75wt% of the GPPMs. Furthermore, the poorly porous carbon membrane of HNDCM-2,000–1,000 is dominantly amorphous with graphitic domains that only surround the pores (Supplementary Fig. 20). This result implies that the graphitization may be facilitated by the highly porous precursors at temperatures lower than those required for nonporous or poorly porous ones, which is a phenomenon that most likely arises from the abundant high-energy surfaces in the porous structures. The graphite sheets are not stable on size shrinkage and tend to rearrange into concentric graphitic shells due to the beneficial effect of symmetric and uniform strain distribution^30^. Furthermore, higher temperatures reduced the diameter of the hollow cages and introduced more shells in the onion-like structures of HNDCMs (Supplementary Figs 21 and 22), which is consistent with previous reports^30^,^31^. Based on these analyses, the observed unique graphitic order in the HNDCM-100,000–900/1,000 samples resulted from the porous precursor that facilitated migration and recrystallization of the carbon atoms into graphite. In fact, our results suggest that the graphitization started at temperature as low as 900 °C. Due to the small and/or thin size of the macropore wall, these graphite sheets in the carbon membrane preferentially rearrange themselves into the lowest energetically state.

### Specific surface area and conductivity characterization

**Figure 3:**
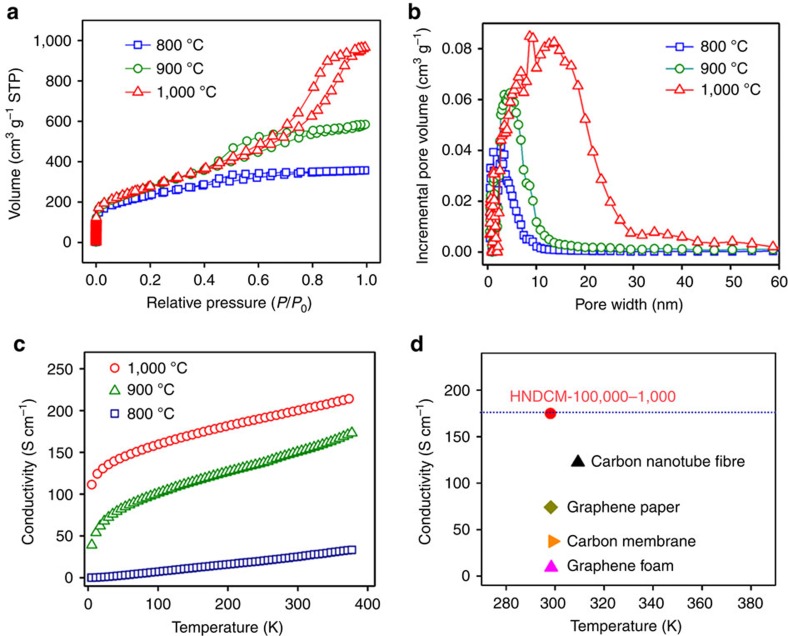
Brunauer–Emmett–Teller specific surface area and conductivity characterizations. (**a**) N_2_ absorption–desorption isotherms and (**b**) corresponding pore size distribution of HNDCM-100,000-1,000/900/800. (**c**) Temperature dependence of the conductivity measured for HNDCM-100,000-1,000/900/800 from 5 to 390 K using a four-probe method. (**d**) Comparison of the conductivity of HNDCM-100,000–1,000 with previous results for macroscopic carbon materials (for example, carbon nanotube fibre^33^, graphene paper^34^, carbon membrane^35^ and graphene foam^36^).

Figure 3a shows the N_2_ absorption–desorption isotherms of HNDCM-100,000-*y* (*y*=800, 900 and 1,000), and the results indicate that the pore volume and specific surface area (S_BET_) significantly increased as the temperature increased. The S_BET_ of HNDCM-100,000–800/-900/-1,000 was 354, 632, and 907 m^2^ g^−1^, respectively, and their total pore volumes were 0.48, 0.61 and 0.79 cm^3^ g^−1^, respectively. The sharp increase of S_BET_ at low pressures (*P*/*P*_0_<0.05) is due to the nitrogen filling in micropores below 2 nm, which is confirmed by the density functional theory pore size distribution curves (Fig. 3b) derived from the N_2_ adsorption branches. The obvious hysteresis above *P*/*P*_0_∼0.5 is indicative of the existence of mesopores. In previous studies, Tf_2_N^−^ in the polymer matrix was reported to act as the micropore forming agent^32^. In our case, Tf_2_N^−^ constitutes 53.8 wt% of GPPM-100,000 based on elemental analysis (Supplementary Fig. 23), and is thereby responsible for the formation of micropores and small mesopores. In these porous membranes with hierarchical architectures, micropores and small mesopores are beneficial and provide active surface areas with high accessibility, and the large mesopores and macropores form interconnected three-dimensional networks and serve as transport highways to accelerate mass diffusion and significantly promote exchange efficiency.

An additional advantage of the highly crystalline graphite structure is the high conductivity of the carbon membranes despite their high pore volume. For example, the conductivity of HNDCM-100,000–1,000 reached its highest value (that is, 200 S cm^−1^) at 298 K, which decreased to 147 and 32 S cm^−1^ at 298 K for HNDCM-100,000–900 and HNDCM-100,000–800, respectively. This high conductivity is appealing for a wide range of electrical/electrochemical applications. Furthermore, the conductivity of HNDCM-100,000-*y* (*y*=800, 900 and 1,000) increased with the test temperature, which is characteristic of semiconductor-like behaviour (Fig. 3c). Notably, the conductivity of HNDCM-100,000–1,000 is one of the highest values ever reported for macroscopic carbon monoliths (Fig. 3d; ref. 33, 34, 35, 36).

### Functionalization and electrochemical performance

Polyelectrolyte-derived complexes can bind and immobilize metal ions, salts and nanoparticles^37^, which inspires us to explore the functionalization of HNDCMs with metal nanoparticles *via* doping the polymeric precursors with metal species. Recently, the conversion of renewable energy resources *via* water splitting to H_2_ and O_2_ is of primary urgency to address issues associated with global warming and energy crisis^38^. Scalable and sustainable electrochemical water splitting is a promising technology. However, this approach requires highly efficient, robust earth-abundant electrocatalyst materials to replace the costly Pt catalyst^39^. To date, remarkable hydrogen evolution reaction (HER) and oxygen evolution reaction (OER) electrocatalysts have been applied in water splitting^40^,^41^. Owing to their thermodynamic convenience and potential applications in proton-exchange membranes or alkaline electrolysers, most efforts in this field have been devoted to developing HER and OER catalysts that function in strongly acidic and basic conditions, respectively^42^,^43^,^44^. However, to accomplish overall water splitting, the coupling of HER and OER catalysts in the same electrolyte is desirable from the viewpoint of simplification of the system and cost reduction^45^,^46^,^47^,^48^,^49^,^50^,^51^,^52^,^53^. Here, the HNDCM-100,000–1,000 sample bearing embedded cobalt nanoparticles (termed HNDDC-100,000-1,000/Co) was investigated as an active bifunctional electrocatalyst for overall water splitting in alkaline media. HNDCM-100,000–1,000/Co was chosen as an example due to its favourable high conductivity and large surface area. Details of the synthesis and structural characterizations are provided in the methods and supporting Information (Supplementary Figs 24–26).

**Figure 4:**
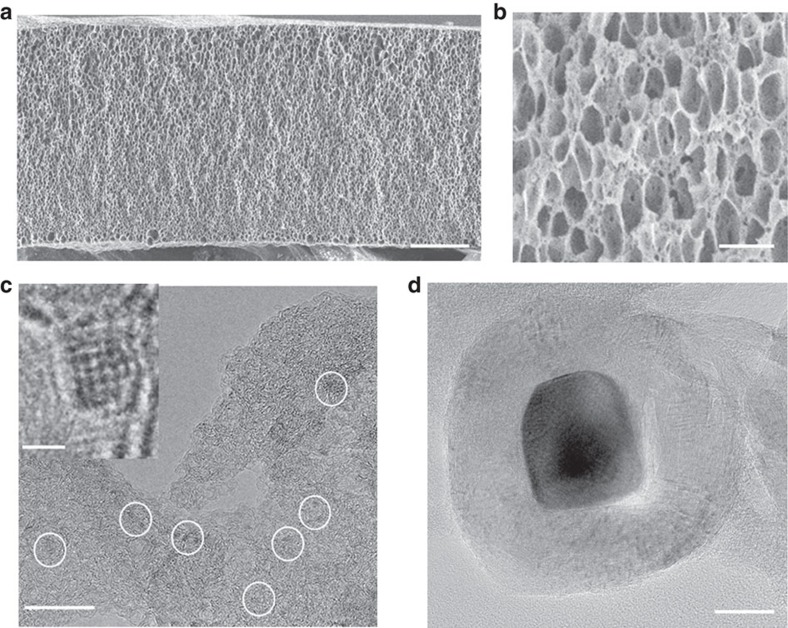
Microstructures of HNDCM-100,000-1,000/Co. (**a**) Cross-section SEM image, scale bar, 20 μm, (**b**) High-magnification SEM image; scale bar, 2 μm, and (**c**,**d**) HRTEM images of HNDCM-100,000-1,000/Co obtained from different areas, scale bar: 10 nm. The ultrafine Co nanoparticles were highlighted with white circles; inset in **c** is the HRTEM image of a single ultrafine Co nanoparticle, scale bar, 1 nm.

The SEM images (Fig. 4a,b) of HNDCM-100,000-1,000/Co suggest that the pore architectures of the HNDCM are preserved during the carbonization in the presence of cobalt acetate. As shown in Fig. 4c,d, after treatment with 1 M aqueous hydrochloric acid (HCl) solution for 12 h, two types of Co nanoparticles were found uniformly distributed throughout the carbon membrane. One type is the ultrafine Co nanoparticles with a diameter of 1.2±0.5 nm (Fig. 4c), and the other is larger Co nanoparticles with a diameter of 20±2 nm covered by a thin graphitic carbon shell of several nm in thickness (Fig. 4d and Supplementary Fig. 26). Previous reports demonstrated that downsizing heterogeneous catalysts in the nanoparticulate form (1-20 nm) could expose more active sites^54^. In addition, it was reported that excess of Co content in N-doped graphene could decrease its HER activity^55^. In accordance with these reports, an etching treatment which removes excessive Co nanoparticles was found to be very beneficial to improve the electrochemical activity and stability, as shown in the case of HNDCM-100,000-1,000/Co.

**Figure 5:**
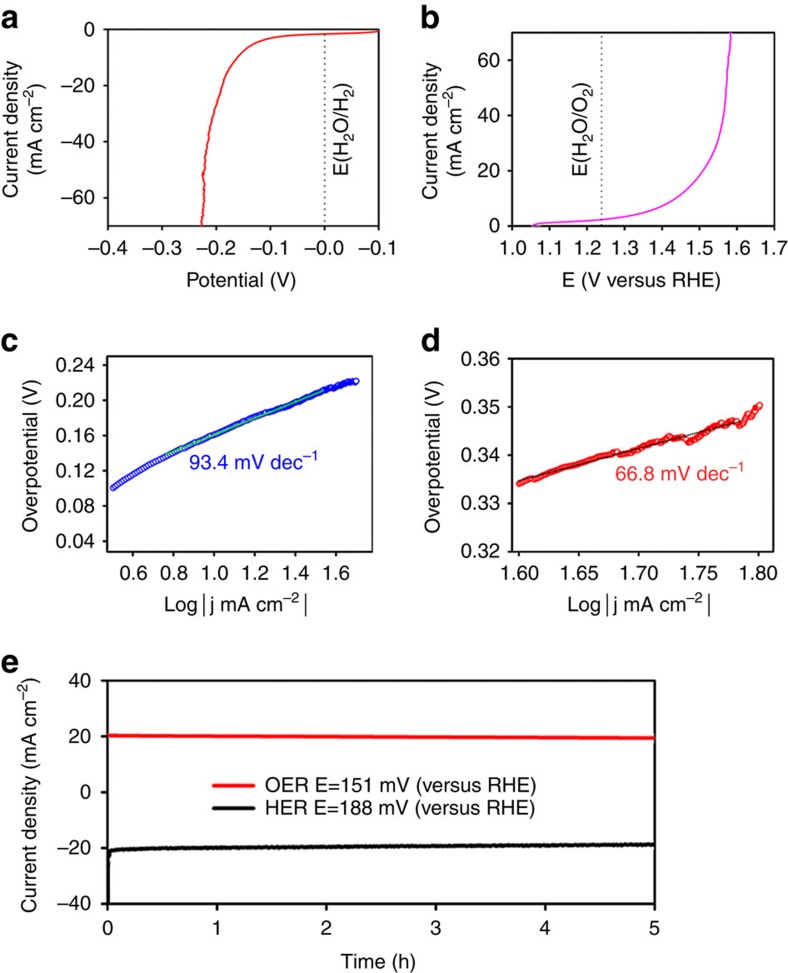
Electrocatalytic performance of HNDCM-100,000-1,000/Co for overall water splitting in 1 M KOH. (**a**,**b**) *J–V* curves after IR correction for HER and OER, respectively; (**c**,**d**) Tafel plots for the data presented in **a**,**b**, respectively; (**e**) Long-term stability test result of the HNDCM-100,000-1,000/Co electrode for HER and OER at a constant voltage.

The electrocatalytic performance of HNDCM-100,000-1,000/Co was evaluated in 1 M KOH solution for both HER and OER. Figure 5a shows the polarization curves obtained from linear sweep voltammetry (LSV) measurements, and a slow sweep rate of 1 mV s^−1^ was used to eliminate any capacitance effect (see experimental detail in supporting information). HNDCM-100,000-1,000/Co exhibited a high HER activity with a current density of 10 mA cm^−2^ at an overpotential of 158 mV after IR correction (the LSV data without IR correction are provided in Supplementary Fig. 27), which is significantly lower than that previously reported for Co/N-doped carbon nanotube catalyst^56^. In addition, this result is comparable or even superior to many other non-noble metal catalysts (Supplementary Table 2). The Tafel slope extracted from the LSV curve was determined to be 93.4 mV dec^−1^ (Fig. 5c), indicating that the HER driven by this catalyst was controlled by a Volmer-Heyrovsky mechanism^57^. Figure 5b shows the LSV curve for the OER. Here, a low overpotential of 199 mV was required to reach a current density of 10 mA cm^−2^, and the Tafel slope was as small as 66.8 mV dec^−1^ (Fig. 5d), close to the ideal value of 59 mV dec^−1^ (equivalent to 2.3RT/F) associated with a one-electron transfer before the rate-limiting step^58^. These values outperform previous results on the Co or CoO_x_/carbon hybrid OER catalysts^59^,^60^. It is important to note that the Co loading in our catalyst was 2.16 wt% (determined by inductively coupled plasma-atomic emission spectroscopy), and therefore, the high catalytic activity of our catalyst for HER and OER is believed to result from a synergy between its high conductivity, nitrogen doping, hierarchical pore architecture and high dispersion state of the active Co nanoparticles in HNDCM-100,000-1,000. Most importantly, unlike previously reported HER electrocatalysts^61^, no performance degradation was induced by bubble trapping in our catalyst due to the rapid mass transfer throughout the hierarchical pore architectures as well as the bubble-repelling surfaces of the nanostructures^62^. The ‘noise' in LSV curves for HER and OER was generated by perturbations in our membrane catalyst due to the release of large amounts of H_2_ and O_2_ bubbles that were produced at higher overpotentials. In general, for powder catalysts, polymer binders are used to process the catalyst films onto conductive substrates, which was avoided in our binder-free carbon membrane. Moreover, vigorous gas production can typically delaminate the active materials from the electrodes due to weakening of the binder, resulting in instability during their long-term operation. In contrast, our free-standing membrane catalyst is free of any polymer binder, leading to high stability for HER and OER (Fig. 5e). Meanwhile, the cyclic voltammetry (CV) durability tests of the 100,000-1,000/Co electrodes for HER and OER were carried out (Supplementary Figure 28). Compared with the long-term stability test of HNDCM-100,000-1,000/Co electrode for HER and OER at a constant voltage, the CV stability data show that the 100,000-1,000/Co electrodes can suffer slightly from a long-time reduction/oxidation process (Supplementary Fig. 28a). In the OER CV durability test data, there is a detectable decrease with time (Supplementary Fig. 28b), which could be attributed to the irreversible reactions occurring at high potentials.

## Discussion

Our experimental results present a viable route towards preparing freestanding, nanoporous carbon membranes that feature an unusual single-crystal-like graphitic order and hierarchical pore architecture as well as favourable nitrogen doping. It was found that polymer precursors of moderate MW and the cross-linking state of polyelectrolyte membranes are crucial to achieve morphology-maintaining carbonization. Owing to the ionic character of the polyelectrolyte membranes and their nature of absorption and immobilization of metal ions, carbon membranes loaded with Co nanoparticles can be readily prepared through carbonization of polyelectrolyte membranes that absorbed cobalt ions. These hybrids are highly active non-noble metal electrocatalyst for overall water splitting.

Importantly, the synthesis and engineering of our membrane-like catalyst can be easily scaled up in size and quantity. As a proof-of-concept demonstration for solar-driven electrolysis, we used a commercially available 20 W solar panel to perform the HER on a piece of HNDCM-100,000-1,000/Co film that was as large as 10.5 × 3.5 cm^2^ (Supplementary Fig. 29), which is the maximum size limited by our carbonization oven. At a non-regulated output voltage of 20 V, an actual H_2_ production rate of ∼16 ml min^−1^ was achieved (Supplementary Fig. 30). This result indicates that our low-cost catalyst meets the requirements for industrial H_2_ production in a large, clean manner in alkaline media. It is important to note that the OER is not only essential for water splitting but is also relevant for the charging process of rechargeable metal-air batteries^63^. The excellent OER activity in combination with the devisable shapes of our membrane-like catalyst affords a new avenue for the development of other efficient energy conversion devices. Furthermore, we expect that the electrocatalytic properties of the HNDCM-based hybrids can be further optimized by choosing appropriate metal species, and also thickness of the carbon membrane. The gradient pore architecture of the porous carbon membrane could offer an ideal platform for exploiting potential applications, such as electro-assisted separation and alternative energy conversion schemes.

## Methods

### Materials and reagents

1-Vinylimidazole (Aldrich 99%), 2,2′-azobis(2-methylpropionitrile; AIBN, 98%), bromoacetonitrile (Aldrich 97%) and bis(trifluoromethane sulfonyl)imide lithium salt (Aldrich 99%) were used as received without further purifications. DMSO, dimethyl formamide, methanol and tetrahydrofuran were of analytic grade. Several PAA samples (MW: 2,000 g mol^−1^, solid powder; MW: 100,000 g mol^−1^, 35 wt% in water, MW: 250,000 g mol^−1^, 35 wt% in water; MW: 450,000 g mol^−1^, solid powder; MW: 3,000,000 g mol^−1^, solid powder ) were obtained from Sigma Aldrich. Poly[1-cyanomethyl-3-vinylimidazolium bis(trifluoromethanesulfonyl)imide] (PCMVImTf_2_N) was prepared according to a previous report^27^.

### Fabrication of hierarchical porous N-doped carbon membrane

First, the as-prepared gradient porous polymer membranes (GPPMs) were clapped between two clean quartz plates and dried at 60 °C overnight under atmospheric pressure. For the carbonization process, the GPPMs were heated to 300 °C at a heating rate of 3 °C min^−1^ under nitrogen flow, and held at 300 °C for 1 h. They were then heated to the targeted carbonization temperature at a heating rate of 3 °C min^−1^ under nitrogen flow. After holding at the final temperature for 1 h, the samples were cooled down to room temperature. During the process of carbonization, the vacuum in furnace was kept constant at 1.5 Torr.

### Fabrication of Co-loaded HNDCM-100,000-1,000 hybrid catalyst

Freshly prepared GPPM-100,000 was placed in 200 ml of cobalt acetate aqueous solution (2 wt%) at pH∼5 adjusted with 0.1 M acetic acid. The mixture was refluxed at 80 °C for 24 h. Afterward, GPPM-100,000-Co(CH_3_COO)_2_ was taken out from the solution, washed with water, and dried at room temperature till constant weight. Finally, pyrolysis of GPPM-100,000-Co(CH_3_COO)_2_ was carried out similarly to that of the HNDCMs, leading to HNDCM-100,000-1,000/Co. Before electrochemical test, the HNDCM-100,000-1,000/Co membranes were immersed in 1 M HCl solution for 12 h to remove exposed Co nanoparticles sitting on the eternal surfaces of the porous carbon membranes.

### Electrochemical measurements

The electrochemical measurements were performed in a conventional three electrode electrochemical cell using a CHI750E station. A graphite rod and an Ag/AgCl (in saturated KCl solution) electrode were used as the counter and reference electrodes, respectively. To avoid the possible effect of Pt deposition on our electrocatalyst during long-term electrochemical reaction, we selected graphite rod as counter electrode in all of the electrochemical reactions. The working electrode was fabricated by wrapping HNDCM-100,000-1,000/Co free-standing film from one side with high-conductive copper tape, which is connected to a copper wire, and the exposed area of copper wires was covered with hot-melt glue to avoid the direct contact with electrolyte. The electrode area was calculated from its surface area. All the applied potentials are reported as reversible hydrogen electrode potential scale using E (versus reversible hydrogen electrode)=E (versus Ag/AgCl)+0.217 V+0.0591 V*pH after IR correction. Potentiostatic EIS was used to determine the uncompensated solution resistance (Rs). The HER and OER activity of HNDCM-100,000-1,000/Co treated with 1 M HCl was evaluated by measuring polarization curves with LSV technique at a scan rate of 1 mV s^−1^ in 1.0 M KOH (pH 14) solution. The stability tests for the HNDCM-100,000-1,000/Co catalysts were performed using chronoamperometry at a constant applied overpotential.

### Characterization

^1^H- and solid-state ^13^C-NMR spectra were recorded on a Bruker AVANCE III spectrometer operating at 400 and 100 MHz resonance frequencies, respectively. NMR chemical shifts were measured with respect to tetramethylsilane (TMS) as an external reference. XRD patterns were collected on a Rigaku powder X-ray diffractometer using Cu K_α_ (*λ*=1.541 Å) radiation. To quantitatively calculate the graphitic degree of carbon membranes prepared with different temperatures, we grinded the carbon membranes into fine powders for the XRD test, avoiding any shift of the (002) peak resulted from the unevenness of carbon membranes. XPS data were collected by an Axis Ultra instrument (Kratos Analytical) under ultrahigh vacuum (<10^−8^ Torr) and by using a monochromatic Al K_α_ X-ray source. The adventitious carbon 1 s peak was calibrated at 285 eV and used as an internal standard to compensate for any charging effects. Raman measurements were performed on a Renishaw inVia Reflex with an excitation wavelength of 473 nm and laser power of 100 mW at room temperature. Nitrogen sorption isotherms were measured at −196 °C using a Micromeritics ASAP 2020M and 3020M system. Samples were degassed for 6 h at 200 °C before the measurements. Pore size distribution was calculated using the density functional theory method. Gel permeation chromatography (GPC) was conducted at 25 °C in a NOVEMA-column with mixture of 80% acetate buffer and 20% methanol as eluent (flow rate: 1.00 ml min^−1^, PEO standards using RI detector-Optilab-DSP-Interferometric Refractometer). Thermal gravimetric analyses were performed on a Netzsch TG209-F1 apparatus at a heating rate of 10 °C min^−1^ under N_2_ flow. Elemental analyses were obtained from the service of Mikroanalytisches Labor Pascher (Remagen, Germany). A field emission scanning electron microscope (FESEM, FEI Quanta 600FEG) was used to acquire SEM images. Transmission electron microscope (TEM) and high-resolution TEM (HRTEM) images, selected-area electron diffraction (SAED) patterns, and the HAADF-STEM-EDS data were taken on a JEOL JEM-2100F TEM operated at 200 kV.

### Data availability

The authors declare that the data supporting the findings of this study are available within the article and its Supplementary Information File or from the corresponding authors upon request.

## Additional information

**How to cite this article:** Wang, H. *et al*. Synthesis of single-crystal-like nanoporous carbon membranes and their application in overall water splitting. *Nat. Commun.* **8**, 13592 doi: 10.1038/ncomms13592 (2017).

**Publisher's note:** Springer Nature remains neutral with regard to jurisdictional claims in published maps and institutional affiliations.

## Supplementary Material

Supplementary Information Supplementary Figures 1-30, Supplementary Tables 1-3 and Supplementary References.
