# Supplementary material for: Synthesis of single-crystal-like nanoporous carbon membranes and their application in overall water splitting
Source: Nat Commun. 2017 Jan 4;8:13592. doi: 10.1038/ncomms13592 (PMC5216123; doi:10.1038/ncomms13592)
Supplement: Supplementary Information — Supplementary Figures 1-30, Supplementary Tables 1-3 and Supplementary References. [file ncomms13592-s1.pdf]

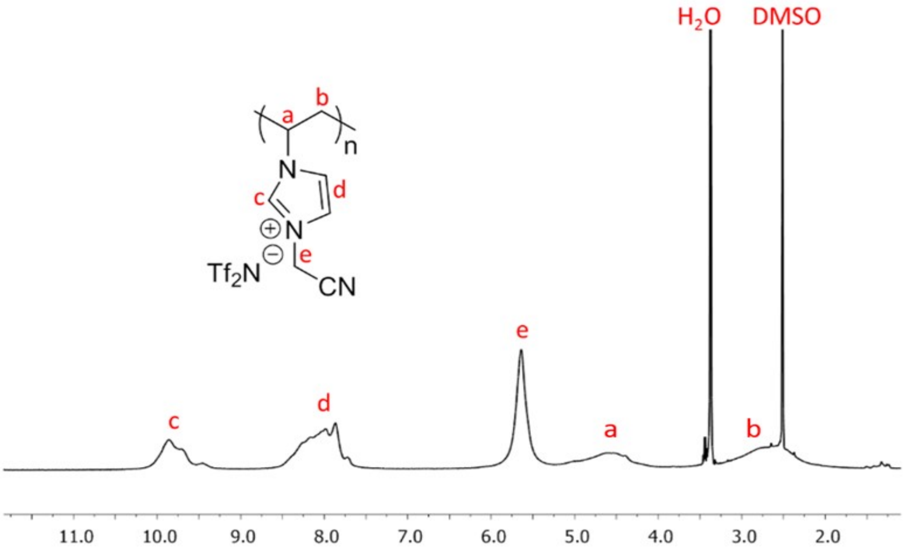

Supplementary Figure 1 | ^{1}H-NMR spectra of PCMVImTf$_2$N in DMSO-d_6.

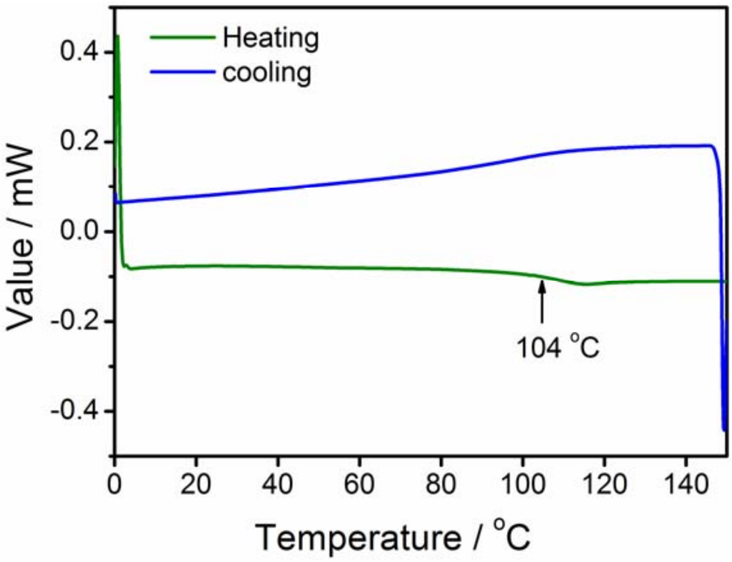

Supplementary Figure 2 | DSC curve of PCMVImTf_2N, showing a glass transition temperature at about 104 ^{o}C.

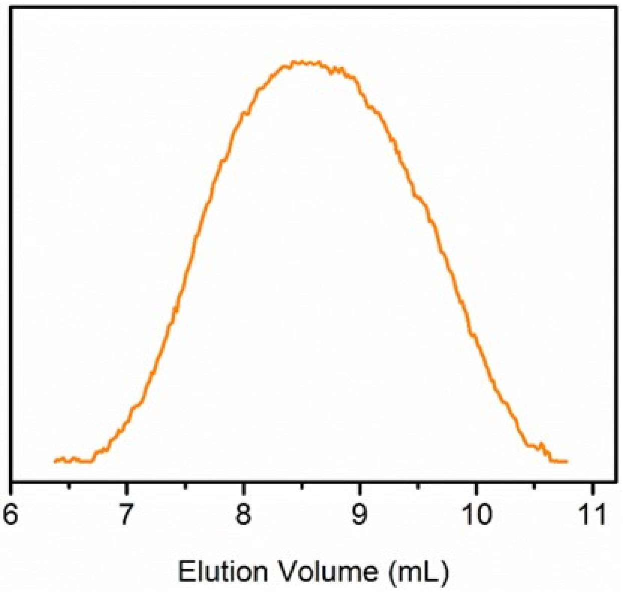

Supplementary Figure 3 | GPC trace measured for PCMVImBr. The apparent number-average molecular weight and PDI value of poly(3-cyanomethyl-1-vinylimidazolium bromide) (PCMVImBr) was measured to be 5.80 × 10^5 g/mol and 3.85, respectively (measured by GPC, eluent: water with a mixture of 80% acetate buffer and 20% methanol). Poly[3-cyanomethyl-1-vinylimidazolium bis(trifluoromethane sulfonyl)imide] (PCMVImTf$_2$N) was prepared by anion exchange of PCMVImBr with LiTf$_2$N salt. Therefore, the apparent number-average molecular weight of PCMVImTf$_2$N is calculated to be 1.12 × 10^6 g/mol.

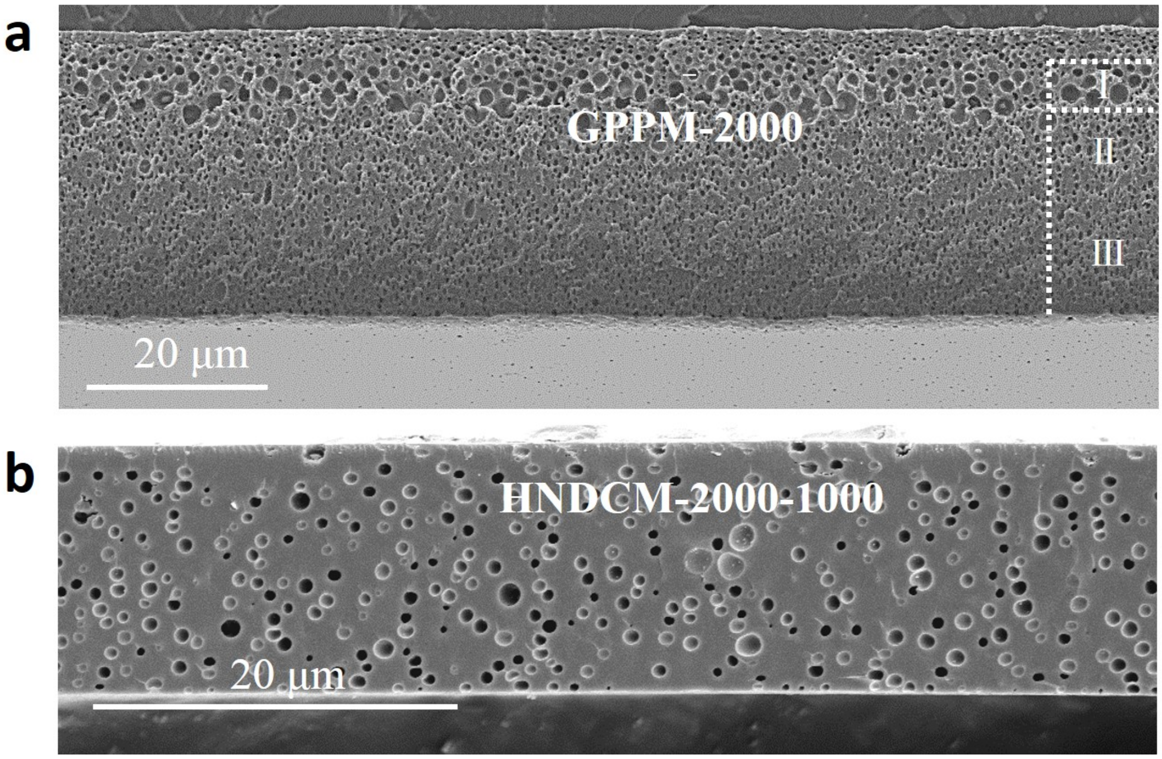

Supplementary Figure 4 | Cross-section SEM images of GPPM-2000 and HNDCM-2000-1000. a, Cross-section SEM image of GPPM-2000 prepared with PCMVImTf$_2$N and PAA of Mw ~ 2000 g/mol. Here, notations of GPPM-x and HNDCM-x-y are used, where x and y denote the Mw of PAA and the carbonization temperature, respectively. **b**, Cross-section SEM image of HNDCM-2000-1000. It clearly shows the pores in GPPM-2000 are continuous in a gradient distribution **(Supplementary Figure 4a)**. The average pore sizes are 2.1 μm in Zone (I), 650 nm in Zone (II), and 600 nm at the bottom, Zone III. From **Supplementary Figure 4b**, it can be clearly seen that the pores in HNDCM-2000-1000 are random and inconsecutive, indicating the morphology-maintaining carbonization can't be achieved by pyrolysis of GPPM-2000.

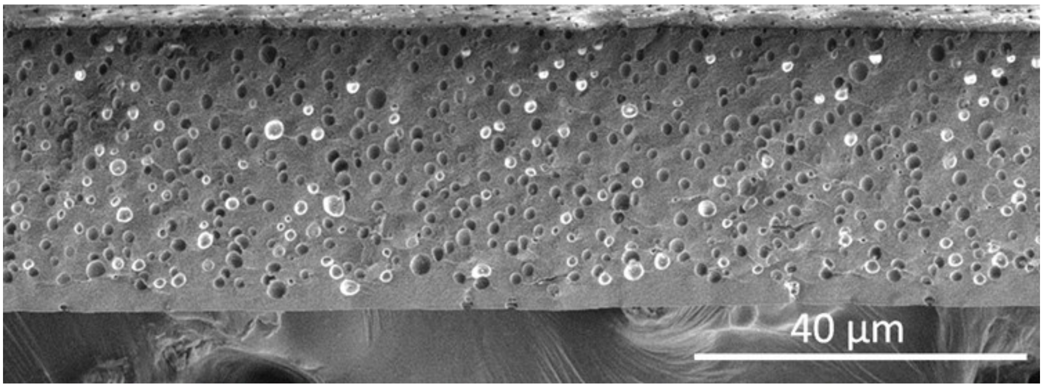

Supplementary Figure 5 | Cross-section SEM image of the carbon sample HNDCM-2000-300 prepared from GPPM-2000 carbonized at 300 ^{o}C.

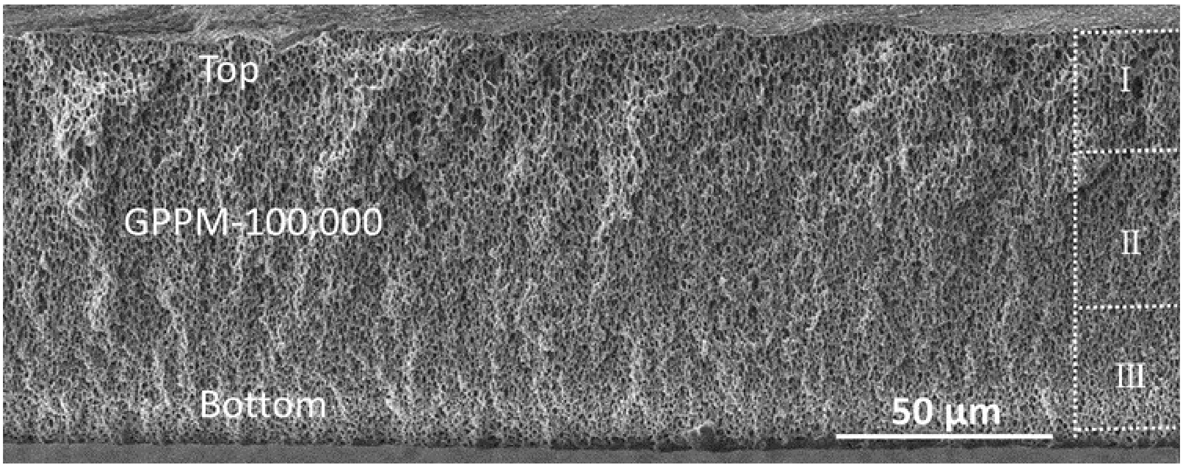

Supplementary Figure 6 | Cross-section SEM image of the GPPM-100,000. The pore sizes gradually decrease from the top layer (zone I, average pore size: 900 nm), to middle layer (zone II, average pore size: 740 nm) and further to bottom layer (zone Ⅲ, average pore ore size: 500 nm).

The formation mechanism of the gradient, hierarchically porous polymer membrane can be explained from a diffusion-controlled kinetic point of view, that is, the diffusion of aqueous NH_3 into the PCMVImTf$_2$N/PAA blend film from the top to the bottom is a crucial step. When the dried PCMVImTf$_2$N/PAA blend film sticking to a glass plate is immersed in aqueous NH_3 solution, rapid and thorough electrostatic complexation takes place in the surface region because of the direct and full contact with the NH_3 solution. After the first stage of full-contact electrostatic complexation, aqueous NH_3 gradually diffuses into the bulk membrane, neutralizes PAA and introduces interpolyelectrolyte complexation. Thus, this diffusion creates a gradient in the degree of electrostatic complexation (DEC) and correspondingly in the pore size distribution. The degree of electrostatic complexation (DEC, defined as the molar fraction of imidazolium units that undergo complexation) of GPPMs prepared with different M_w of PAA are listed in **Supplementary Table 1**.

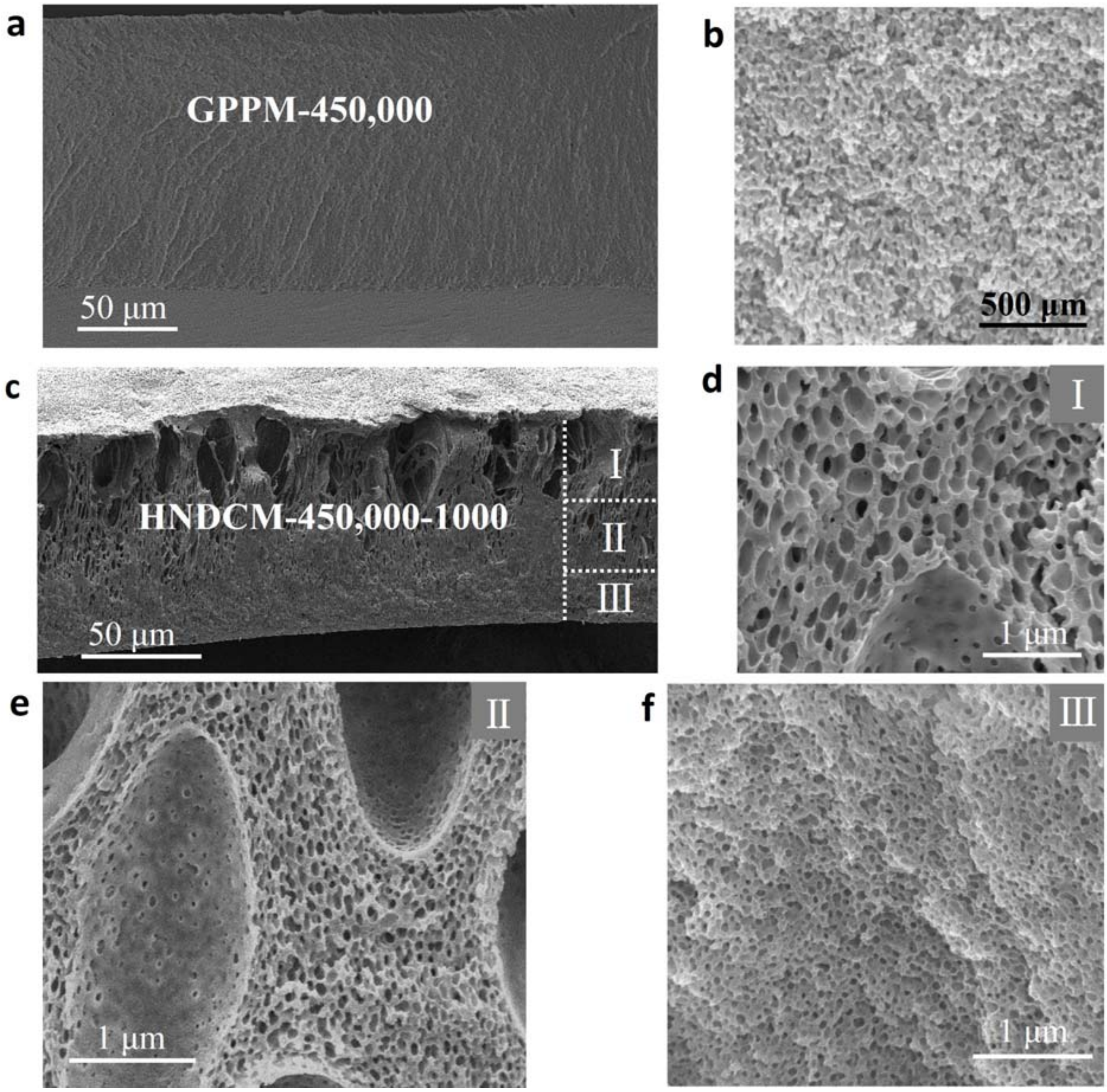

Supplementary Figure 7 | Cross-section SEM images of GPPM-450,000 and HNDCM-450,000-1000. **a**, Cross-section SEM image of GPPM-450,000; **b**, Enlarged SEM image of GPPM-450,000; **c**, Cross-Section SEM image of HNDCM-450,000-1000; **d-f**, Representative SEM images of the cross-section structures in Zone I, II and III of HNDCM-450,000-1000.

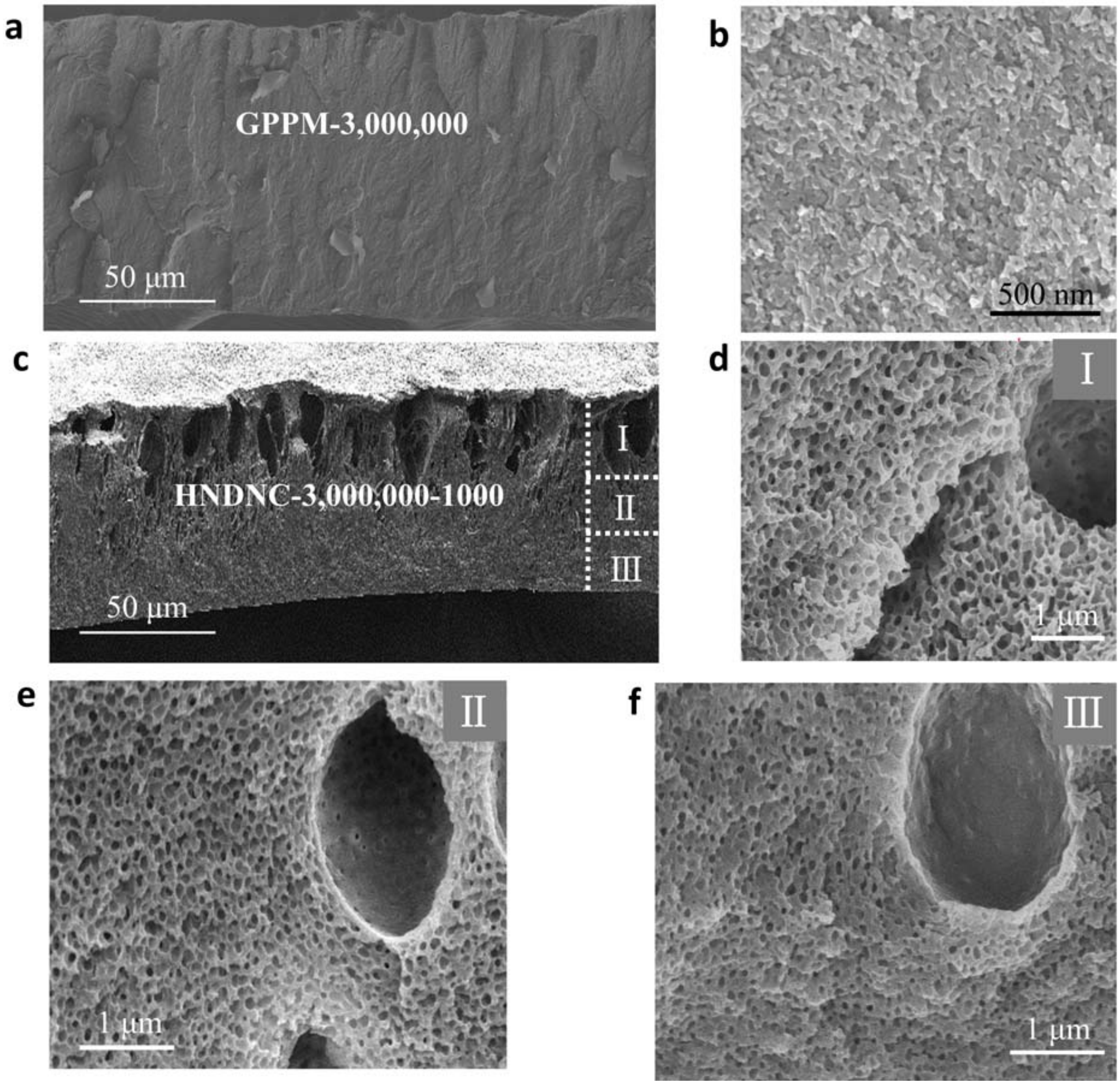

Supplementary Figure 8 | Cross-section SEM images of GPPM-3,000,000 and HNDCM-3,000,000-1000. a, Cross-section SEM image of GPPM-3,000,000; **b**, Enlarged SEM image of GPPM-3,000,000. **c,** Cross-section SEM image of HNDCM-3,000,000-1000; **d-f**, Representative SEM images of the cross-section structures in Zone I, II and III of HNDCM-3,000,000-1000.

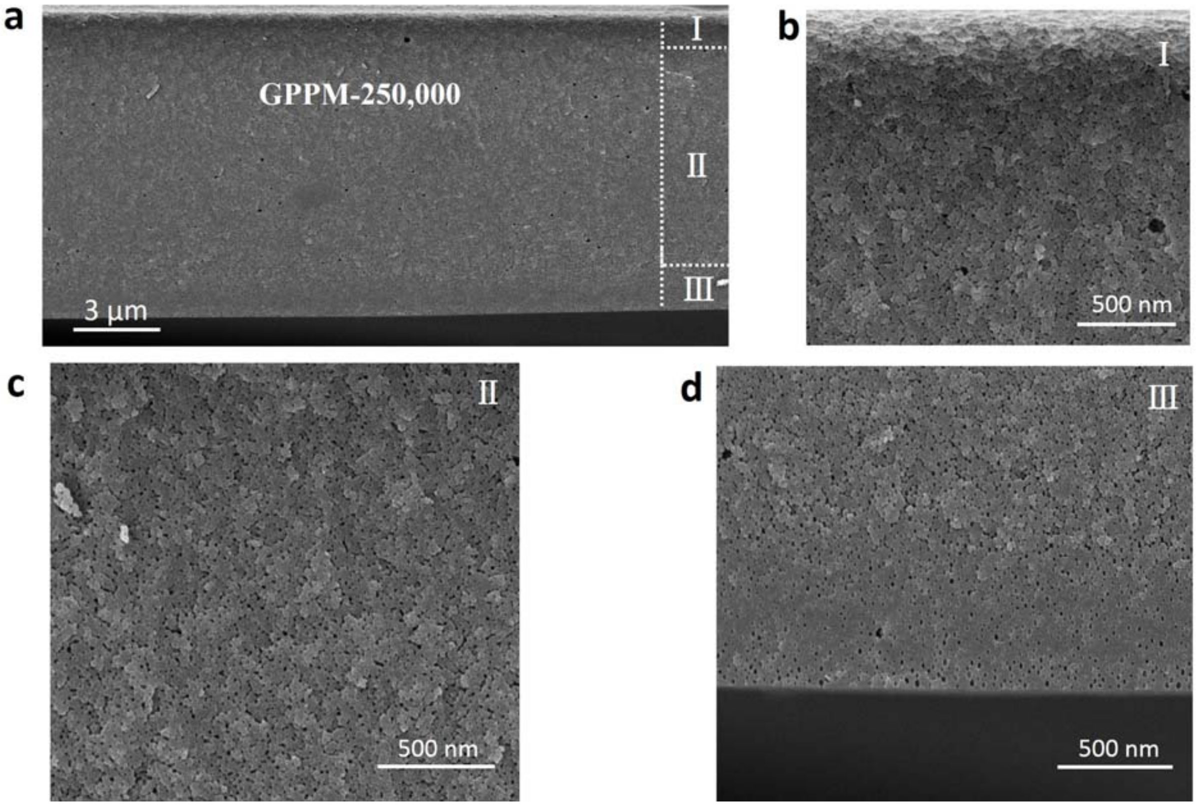

Supplementary Figure 9 | Cross-section SEM images of GPPM-250,000 a, Low magnification SEM image of GPPM-250,000. **b-d**, Representative SEM images of the cross-section structures in Zone I, II and III of GPPM-250,000.

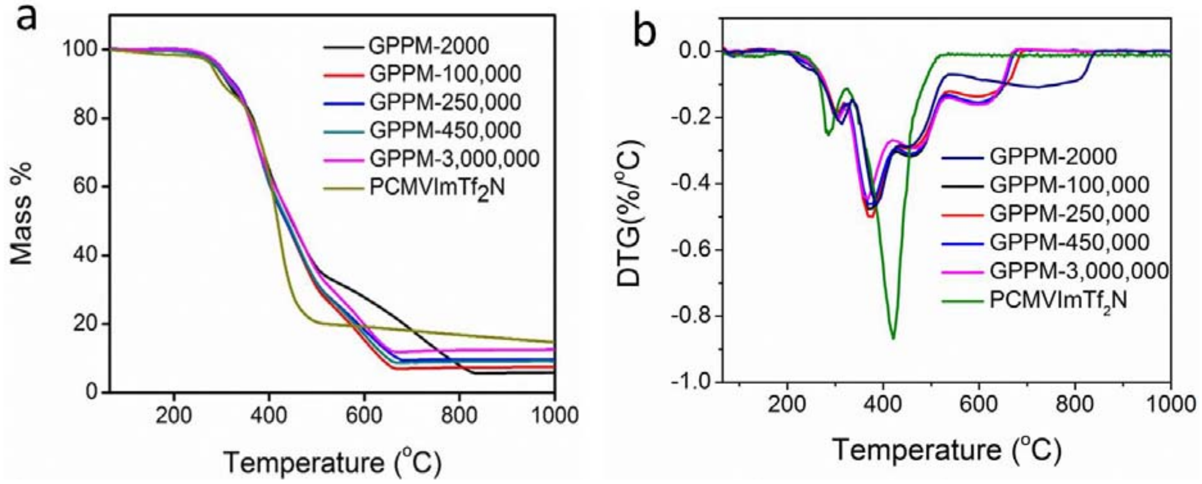

Supplementary Figure 10 |. Thermal analysis of $PCMVImTf_2N$ and GPPMs prepared with $PCMVImTf_2N$ and PAA of different MWs. a, TGA and **b**, DTA curves.

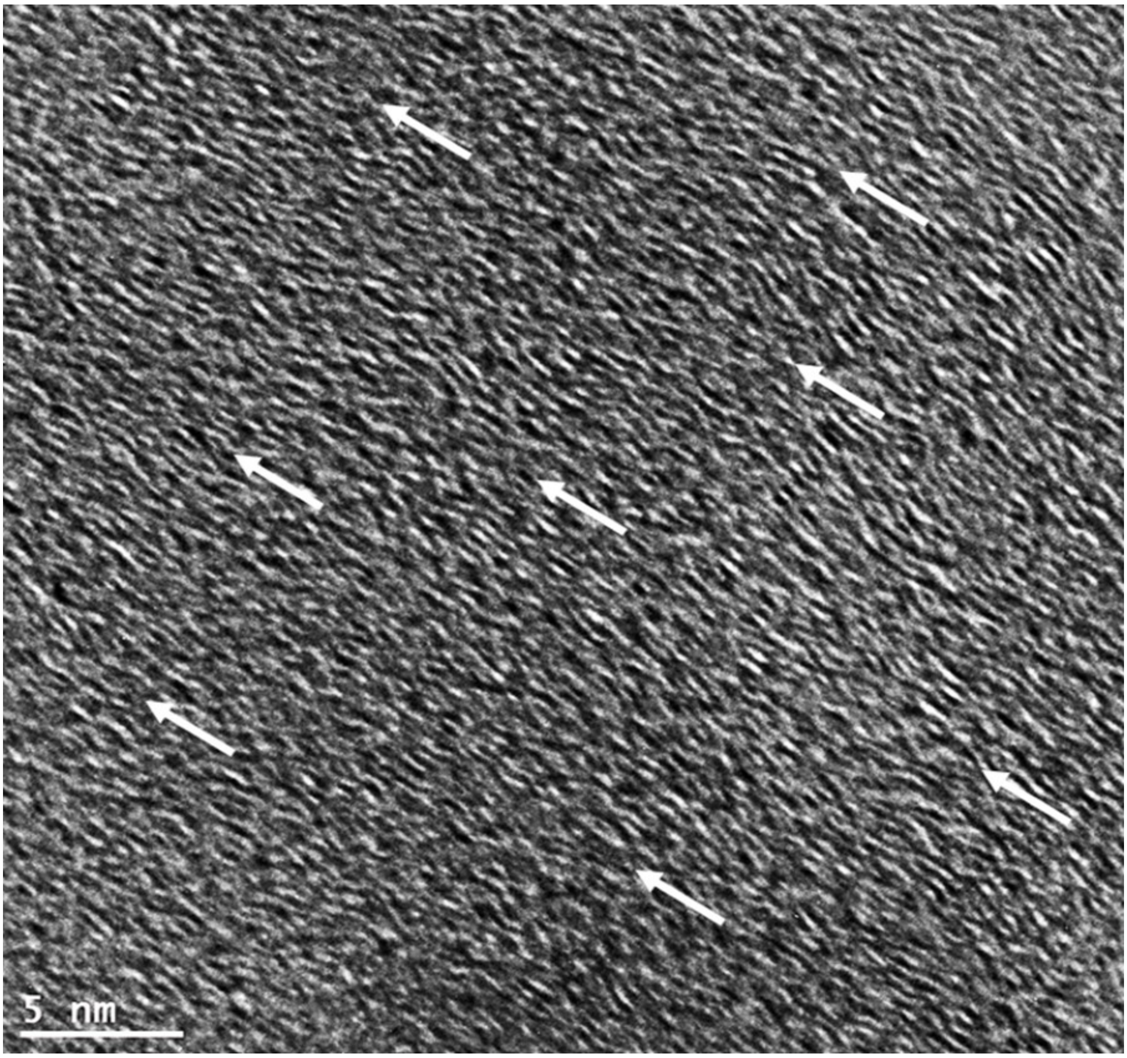

Supplementary Figure 11 | HRTEM image of the HNDCM-100,000-800. The white arrows point out the preferential orientation of the graphitic layers.

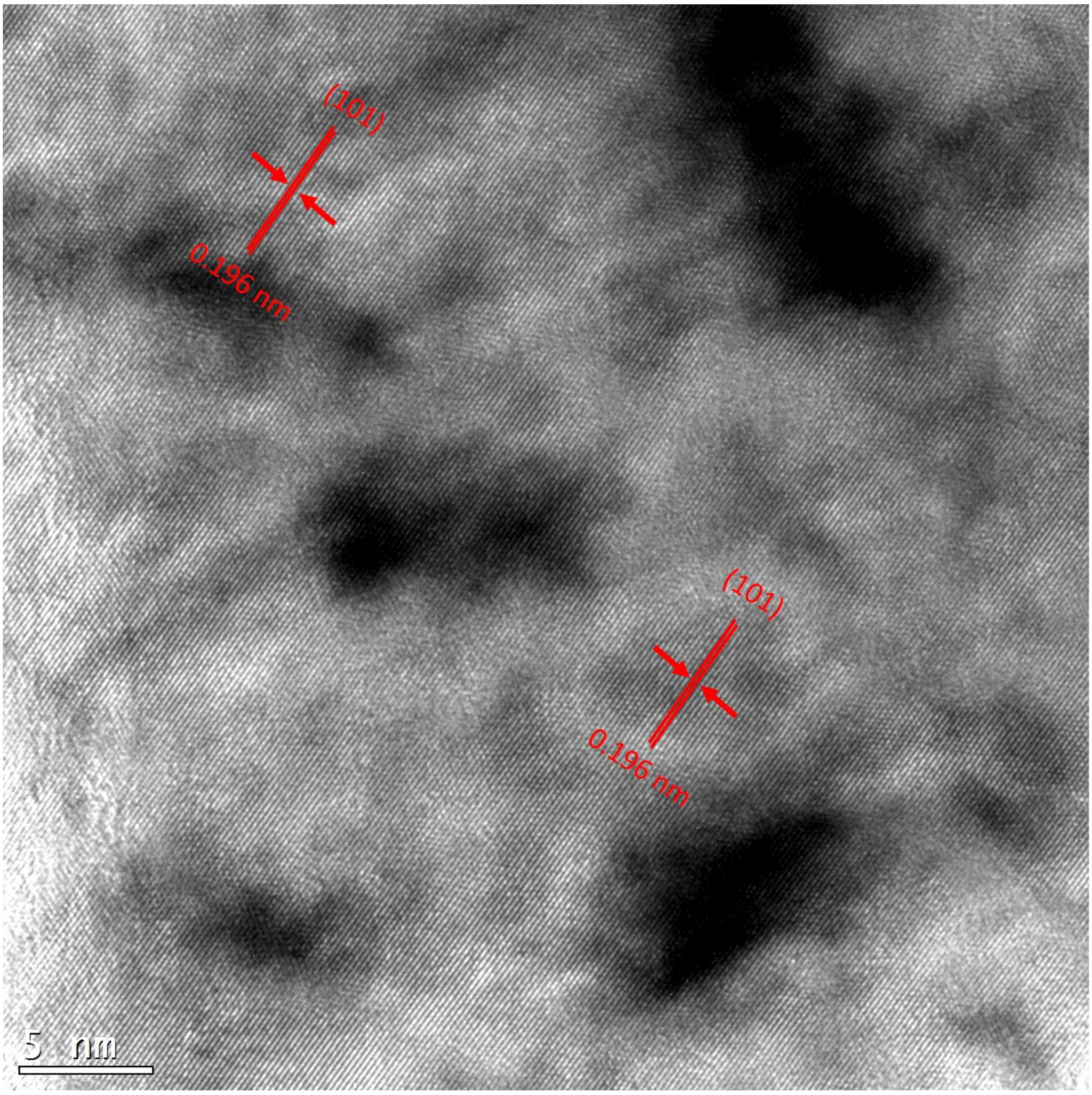

Supplementary Figure 12 | HRTEM image of the HNDCM-100,000-900 with (101) plane dominated sheets. It should be noted that in HNDCM-100,000-900, we observed two phases: one is the (101) plane dominated graphitic sheets in the membrane matrix, as shown here; the second phase is the (002) plane dominated concentric onion-like graphitic structures (HRTEM **Supplementary Figure 21**).

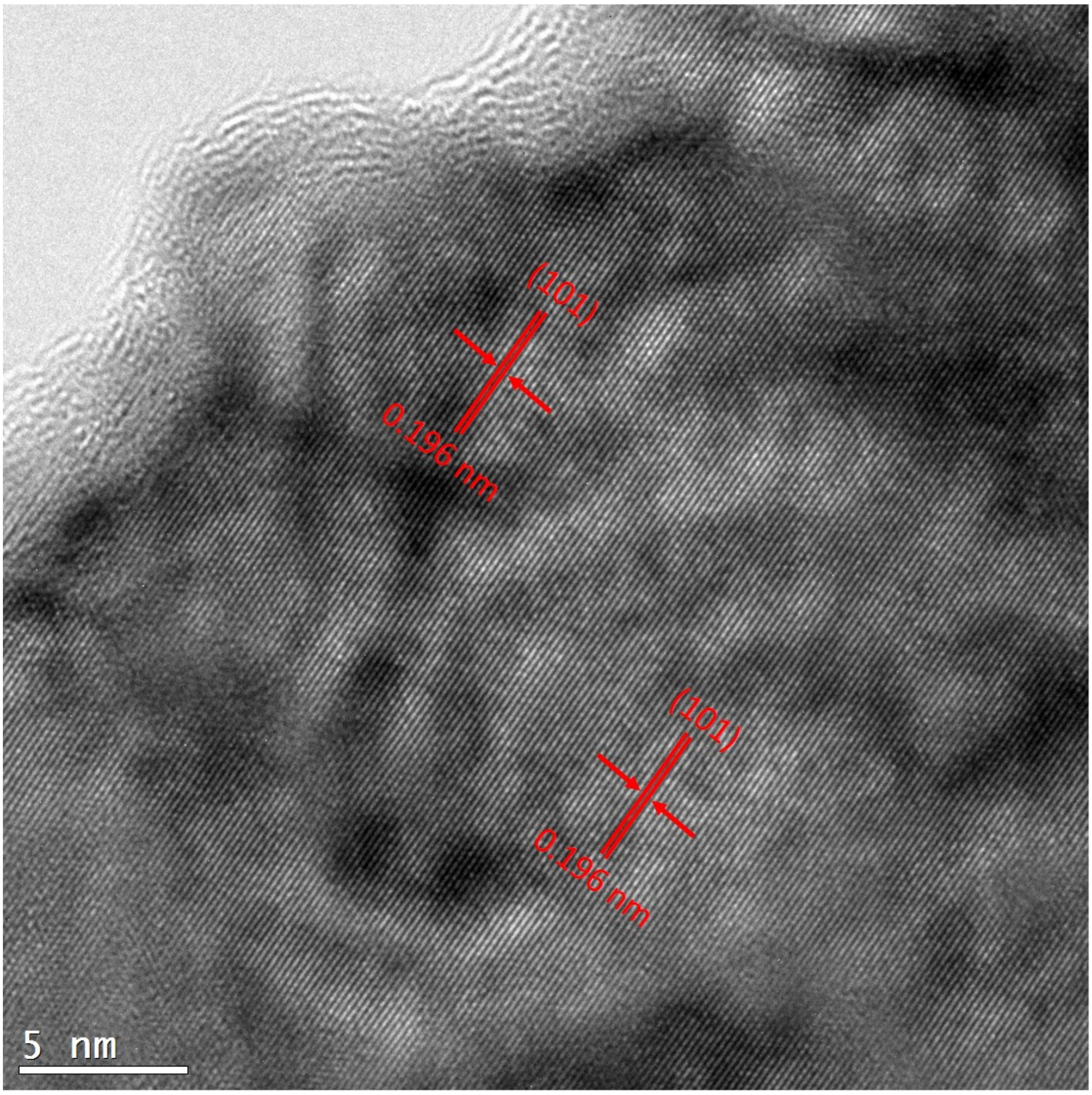

Supplementary Figure 13 | HRTEM image of the HNDCM-100,000-1000 with (101) plane dominated sheets. Similar to HNDCM-100,000-900, in HNDCM-100,000-1000, we also observed two phases: one is the (101) plane dominated graphitic sheets, as shown here; the second phase is the (002) plane dominated concentric onion-like graphitic structures (HRTEM **Supplementary Figure 22**).

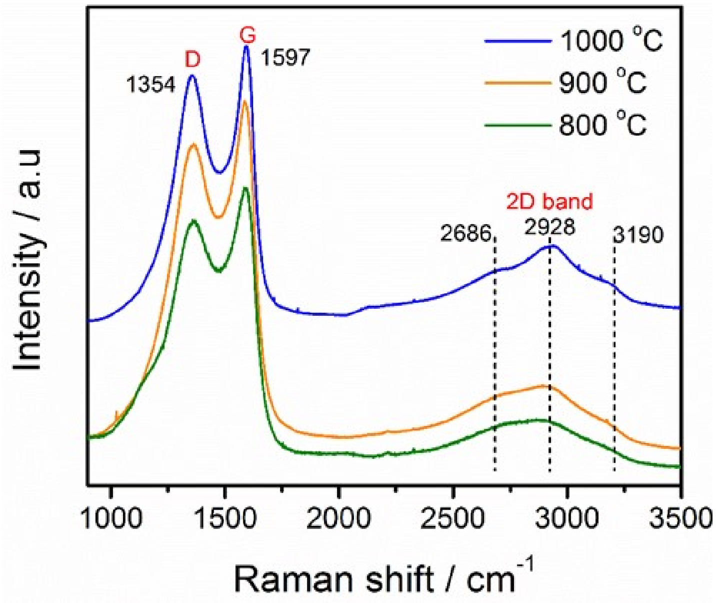

Supplementary Figure 14| Raman spectra of HNDCM-100,000-y (y=800, 900, and 1000). The Raman spectra of the HNDCM-100,000-y samples (y=800, 900, 1000) contain two bands at 1354 and 1597 cm^{-1}, which were assigned to the typical disorder band (D band) and graphitic band (G band) of carbon, respectively. The I_D/I_G ratio for all three samples was ~0.85, indicating their structural similarity[1]. The 2D band is Raman active for crystalline graphitic carbons and sensitive to the π band in the graphitic electronic structure[2]. The 2D peak became much sharper as the carbonization temperature increased, and the most intense peak was observed for HNDCM-100,000-1000.

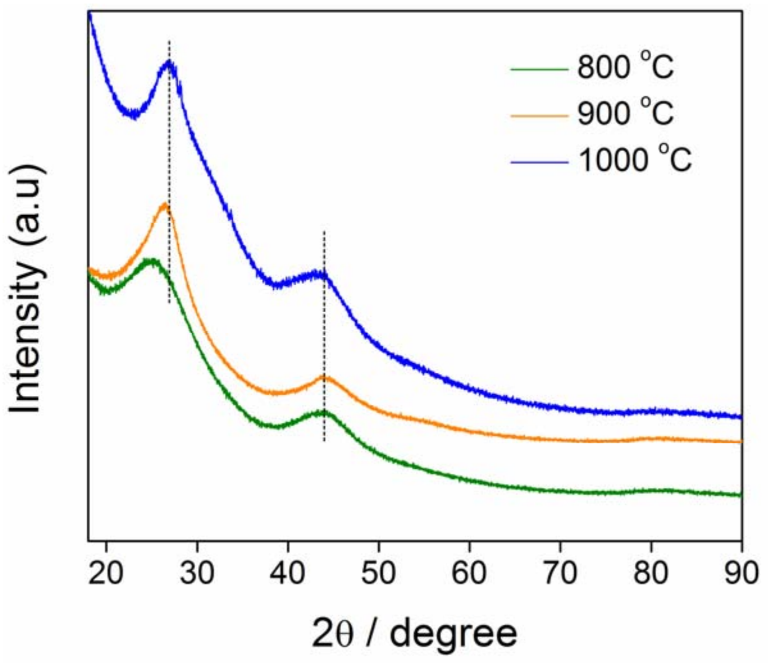

Supplementary Figure 15 | XRD patterns of HNDCM-100,000-y (y=800, 900, and 1000)

We observed the crystalline phase in HNDCM-100,000-900/1000 samples in TEM, which can only reflect local structure information. To obtain a general picture of the crystalline phases of HNDCM-100,000-900/1000, the two samples were further analyzed by X-ray diffraction[3]. The graphitization index can be used to characterize quantitatively the degree of graphitization of carbon materials. The graphitization index is derived from the average interplanar spacing between two successive graphite layers according to the equation 1:

$$g_p = \frac{3.440 - d_{002}}{3.440 - 3.354} \quad \text{.................. Equation 1}$$

According to the Bragg's law, the interplanar spacing is given by $d_{002} = \lambda/\sin\theta$, where λ is the wavelength of the incident X-ray beam. The copper K_α line is 1.541 Å. The θ values of HNDCM-100,000-900 and HNDCM-100,000-1000 are 26.74^o and 26.93^o, respectively. Correspondingly, the d_{002} values of HNDCM-100,000-900 and HNDCM-100,000-1000 are 3.42 and 3.40 Å, respectively. The graphitization index of HNDCM-100,000-900 and HNDCM-100,000-1000 are calculated to be 0.23 and 0.46, respectively. This indicates the degree of crystallinity of carbon membranes is favored expectedly by higher pyrolysis temperature. It

should be mentioned that due to the incorporation of nitrogen atoms into the graphitic phase, the interplaner spacing in nitrogen doped carbons is enlarged to accommodate the lone electron pair on the nitrogen atoms.

In addition to the position, the width of the (002) band provides useful information to estimate the average grain size of graphitic phases. On the basis of the width, the coherence lengths Lc and La can be estimated by the Debye-Sherrer equations:

$Lc = k\lambda / \beta \cos \theta$ .... Equation 2

$La=1.84\ k\lambda /\beta \cos \theta$ .... Equation 3

where k is the shape coefficient, usually k is set to 1. λ is the wavelength of the incident beam, θ is the Bragg angle, and β is the full width at the half maximum (fwhm). The calculated Lc/La values for the HNDCM-100,000-900 and HNDCM-100,000-1000 are 0.44 nm/0.81nm and 0.58 nm/1.06 nm, respectively.

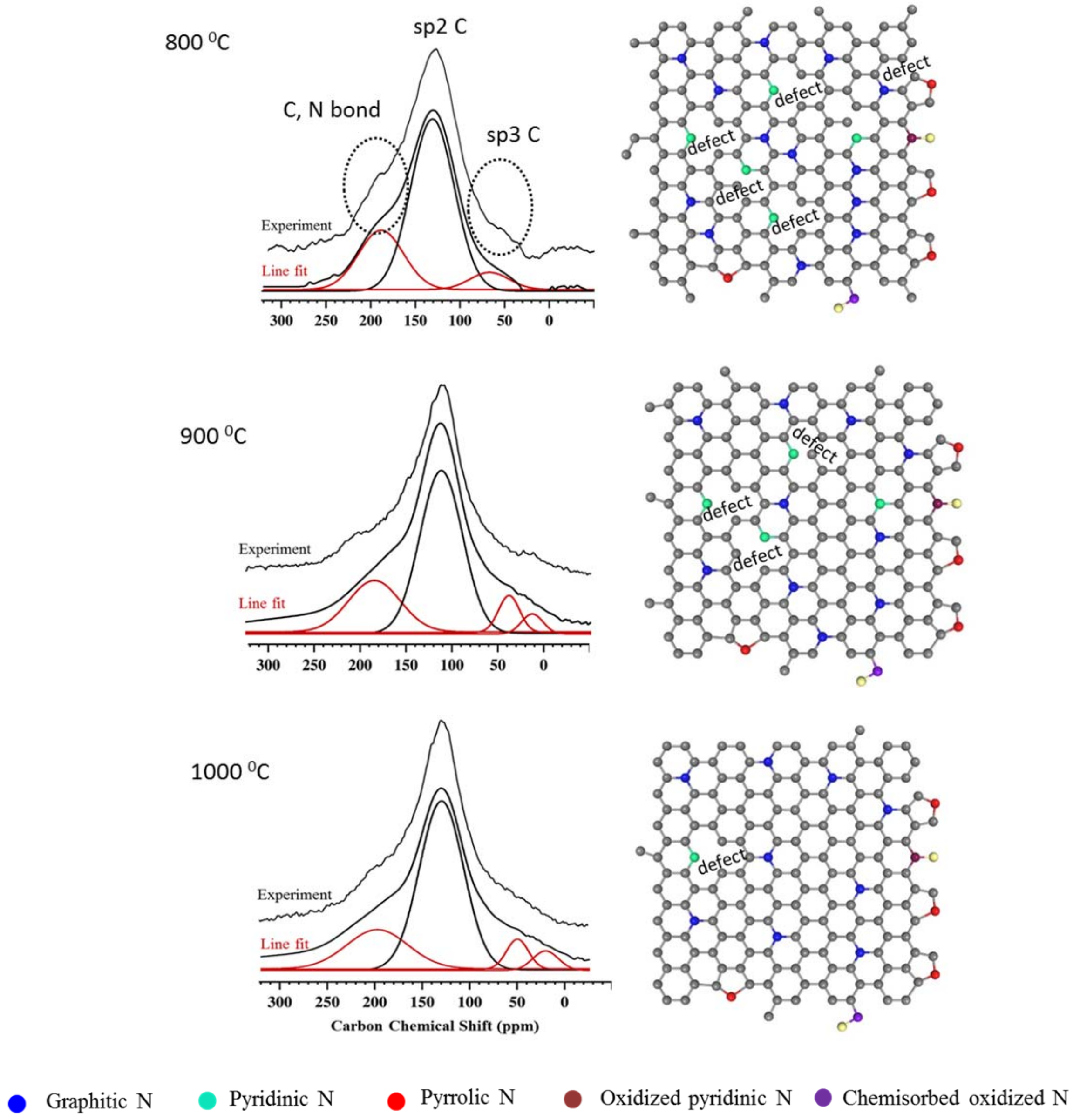

Supplementary Figure 16 | Solid-state ^{13}C-NMR of HNDCM-100,000-y (y=800, 900, and 1000). Solid state ^{13}C-NMR spectroscopies afford the qualitative and quantitative analysis of HNDCM structures. The fitted lines show that the content of carbon bonded nitrogen and sp^3 hybrid C decreases while the content of sp^2 hybrid C increases with increasing carbonization temperature from 800 to 1000 ^{o}C, indicating that higher carbonization temperature can result in higher degree of graphitization. The result that the content of carbon bonded nitrogen decreases with increasing temperature is in agreement with the elemental analysis.

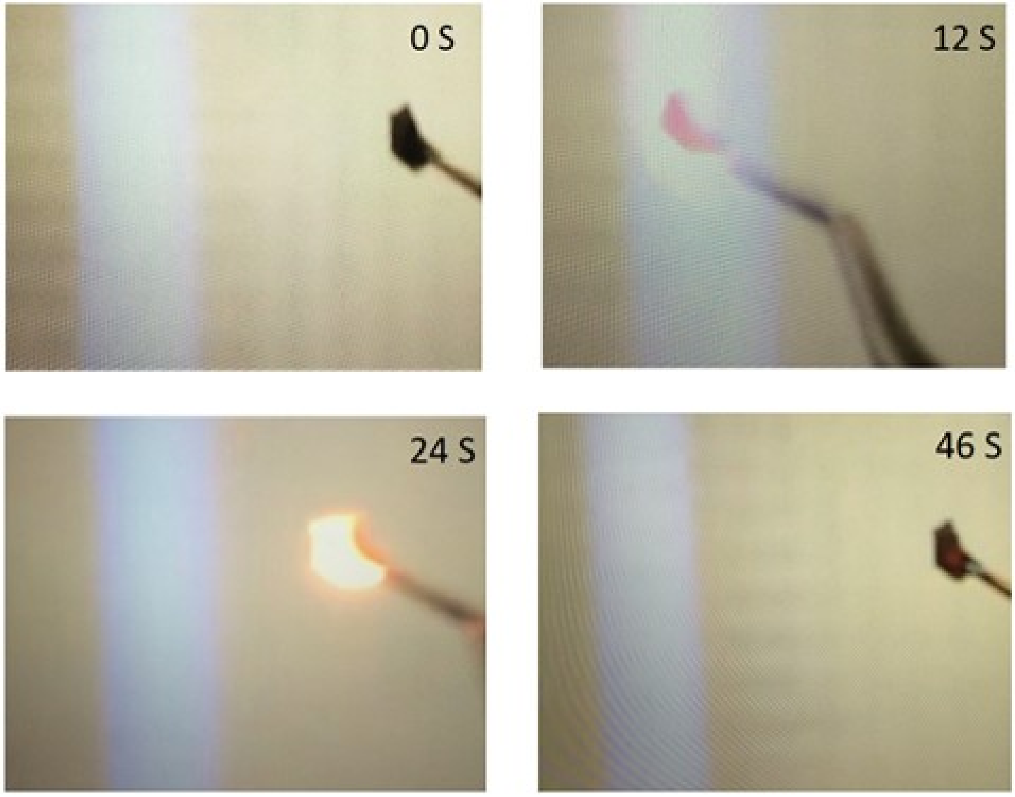

Supplementary Figure 17 | Fire retardant property of HNDCM-100,000-1000. Illustration of the fire retardant property of the nitrogen doped porous carbon membrane by firing the carbon membrane with an acetylene gas burner (flame temperature above 1000 ^{o}C) for 24 seconds. The membrane was found to turn light red in the flame but resumed its native black color 20s after being pulled back to air.

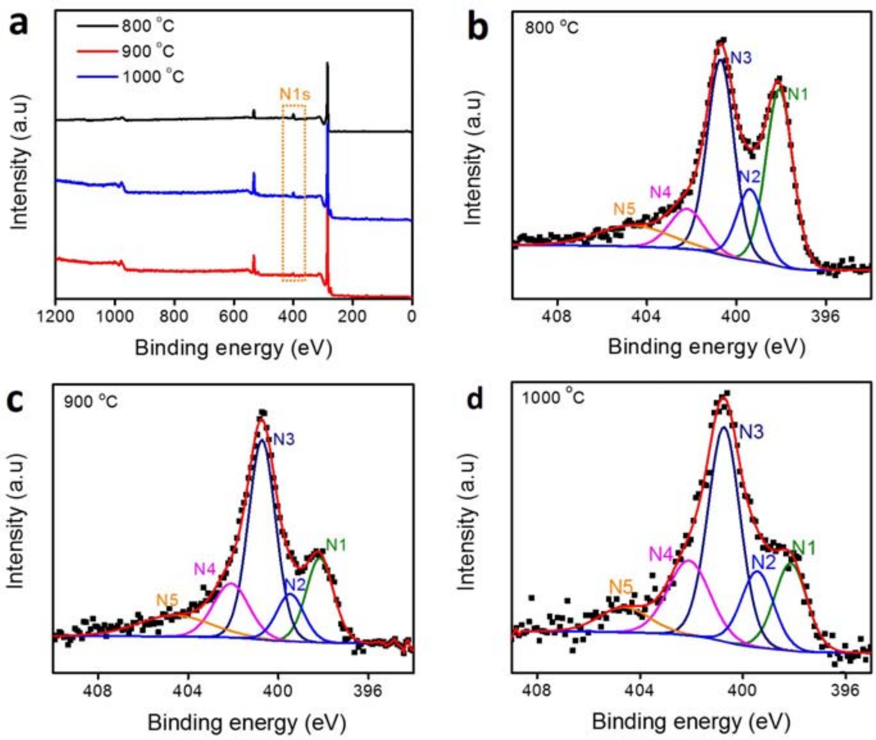

Supplementary Figure 18 | XPS characterization of HNDCM-100,000-y (y=800, 900, and 1000) samples. a, XPS spectra of HNDCM-100,000-y (y=800, 900, and 1000). **b-d**, The fitted XPS peaks for N1s orbit of HNDCM-100,000-y (y=800, 900, and 1000).

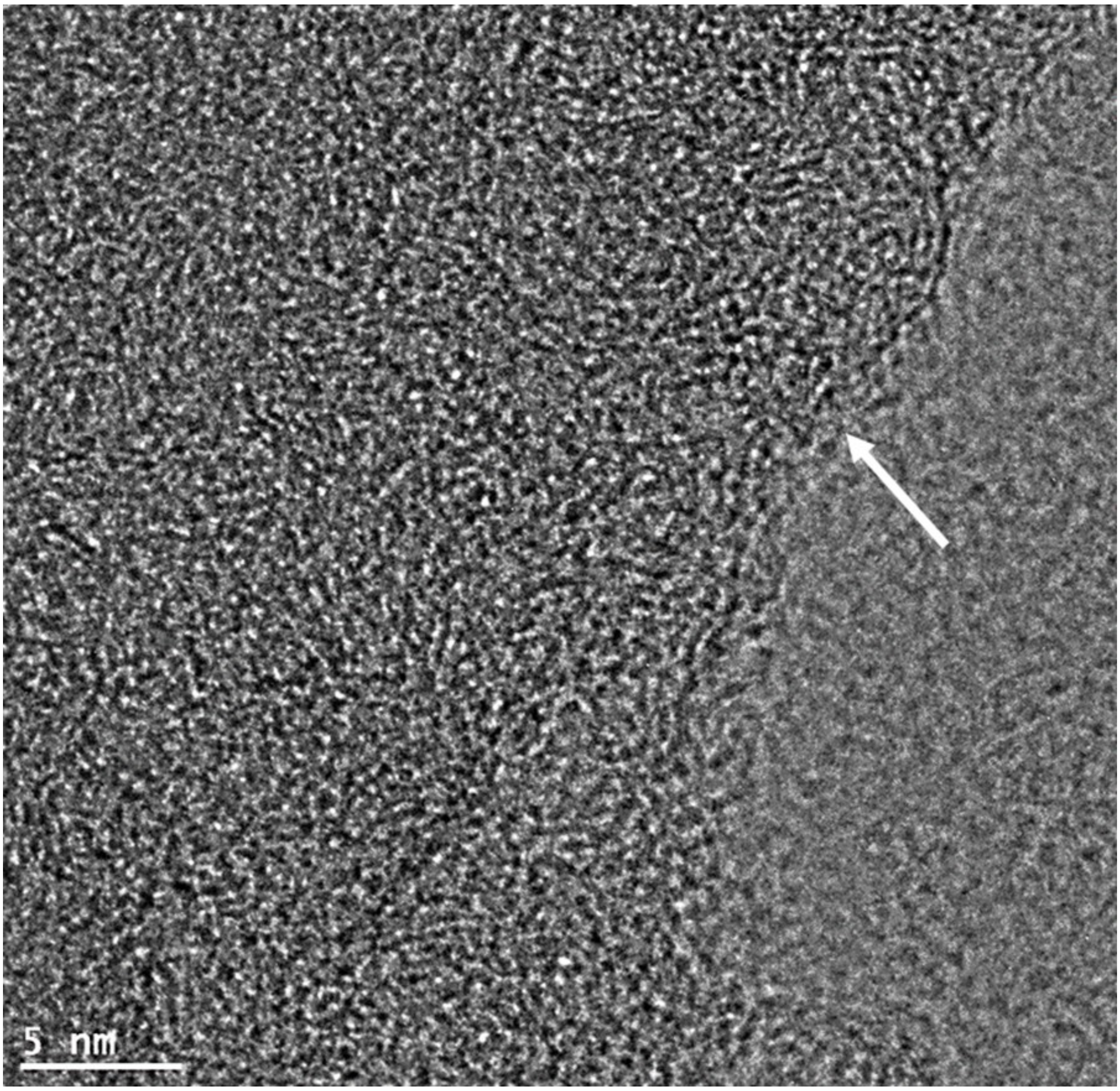

Supplementary Figure 19 | HRTEM of carbon prepared by direct pyrolysis of native nonporous PCMVImTf$_2$N at 1000 ^{o}C. The white arrow indicates the N-doped carbon.

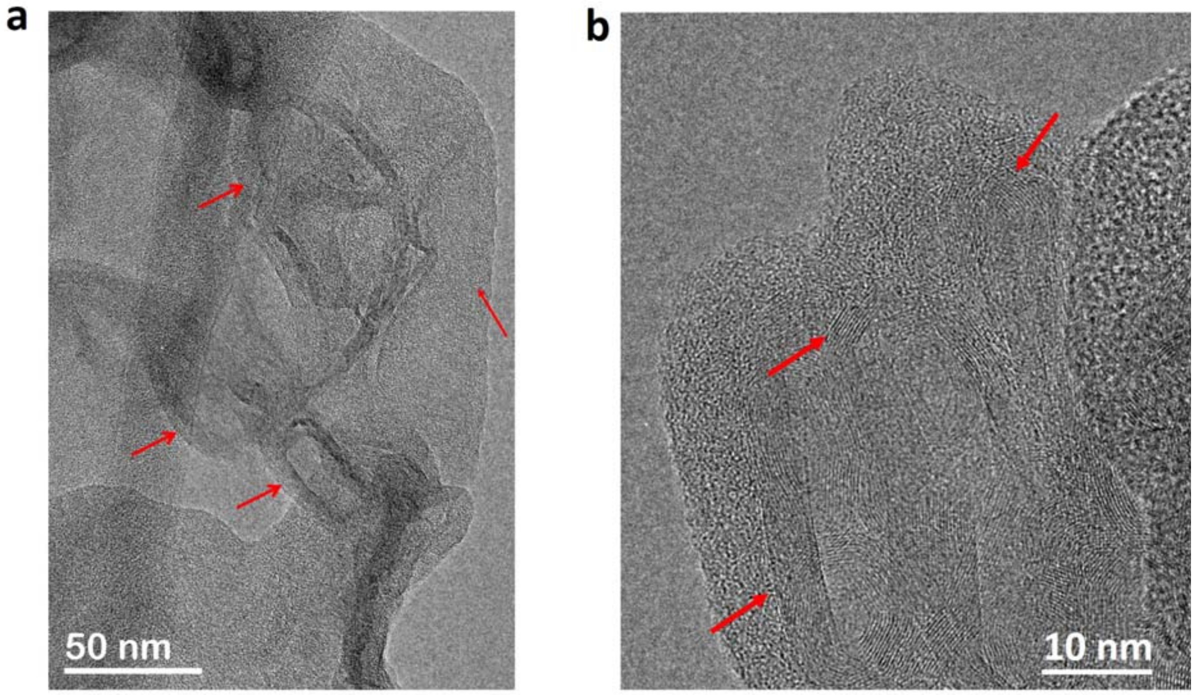

Supplementary Figure 20 | TEM images of the HNDCM-2000-1000. a, Low-magnification TEM image of the HNDCM-2000-1000; **b**, HRTEM image of HNDCM-2000-1000. Red arrows indicate the graphitic N-doped carbon.

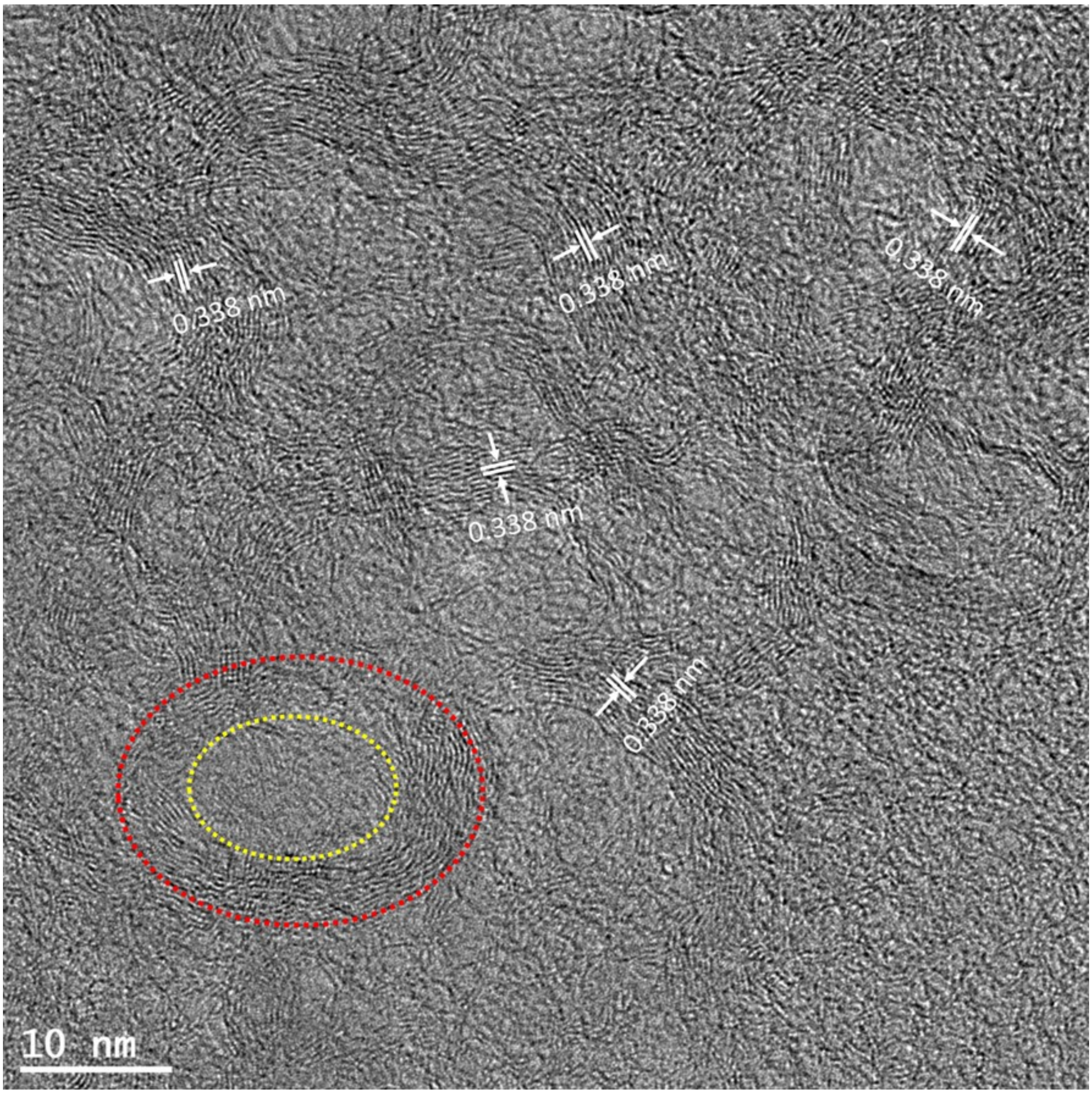

Supplementary Figure 21 | HRTEM image of HNDCM-100,000-900 with (002) plane dominated concentric onion-like graphitic domains. Concentric onion-like graphitic nanostructures with multi-shells (red line) and hollow cages (yellow line) are observable in the HNDCM-100,000-900.

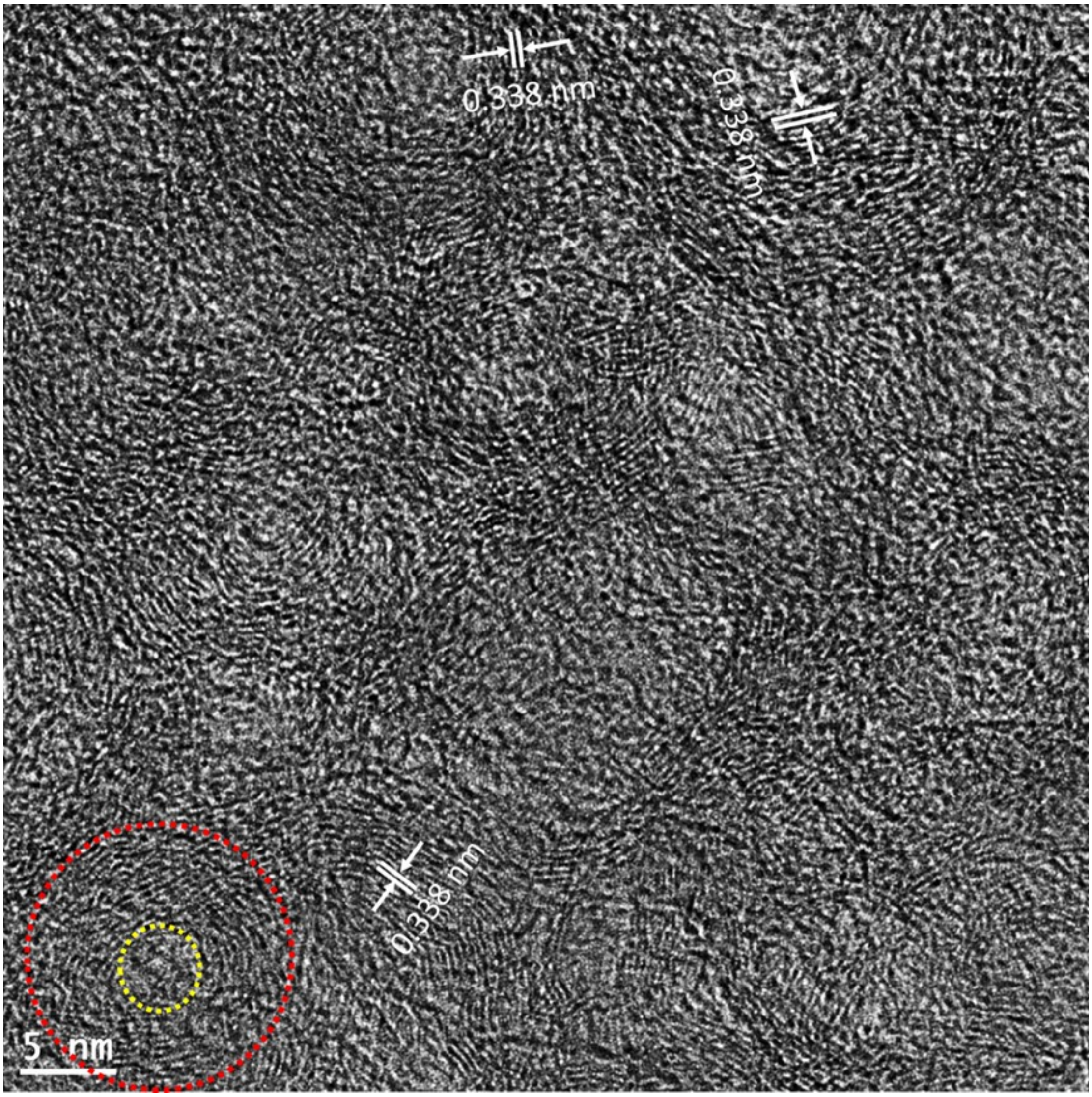

Supplementary Figure 22 | HRTEM image of HNDCM-100,000-1000 with (002) plane dominated concentric onion-like graphitic nanostructures. It can be seen that the typical concentric onion-like graphitic nanostructures with multi-shells (red line) and hollow cages (yellow line) exist in HNDCM-100,000-1000.

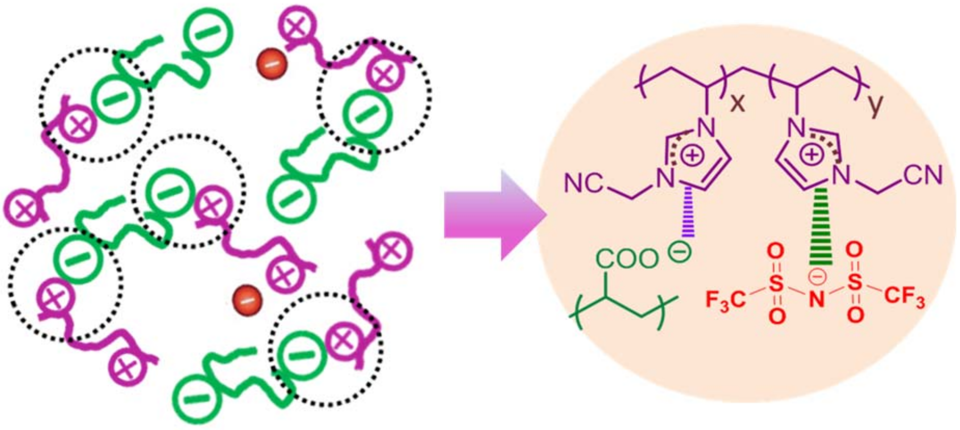

DEC= X/(X+Y) ………………. Equation 4

DEC= (482S-64)/(287S-64) ………………. Equation 5

Supplementary Figure 23 | Scheme to illustrate the definition of the degree of electrostatic complexation (DEC) of the GPPM-100,000-1000.

Equations for defining and calculating DEC. In equation S4, X denotes the imidazolium units that undergo electrostatic complexation with COO^- groups on PAA; Y denotes the imidazolium units in the membrane that are not involved in the electrostatic complexation. In equation S5, S denotes the sulfur weight content. The results of the elemental analyst show that the S content is 12% in GPPM-100,000. We can calculate the DEC of GPPM-100,000-1000 is 20.8%. The content of the bis(trifluoromethanesulfonyl)imide (Tf_2N^-) can be calculated as 28.3 mol % and 53.8% wt% in the GPPM-100,000.

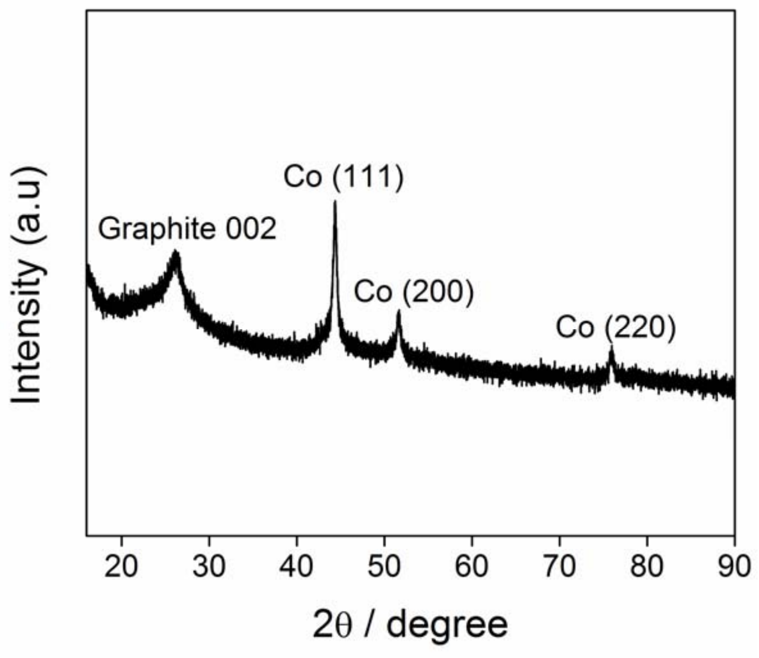

Supplementary Figure 24 | XRD patterns of HNDCM-100,000-1000/Co.

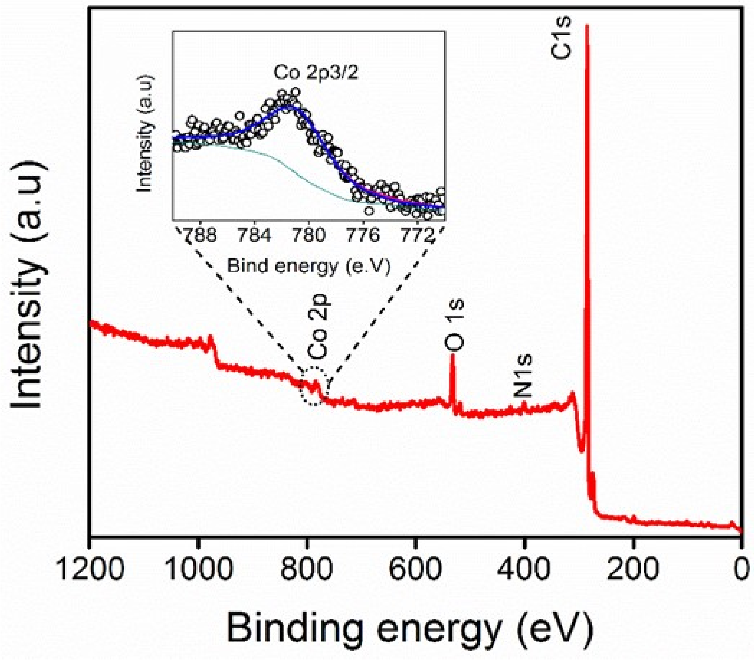

Supplementary Figure 25 | XPS spectra of HNDCM-100,000-1000/Co, inset is the Co 2p3/2 peak.

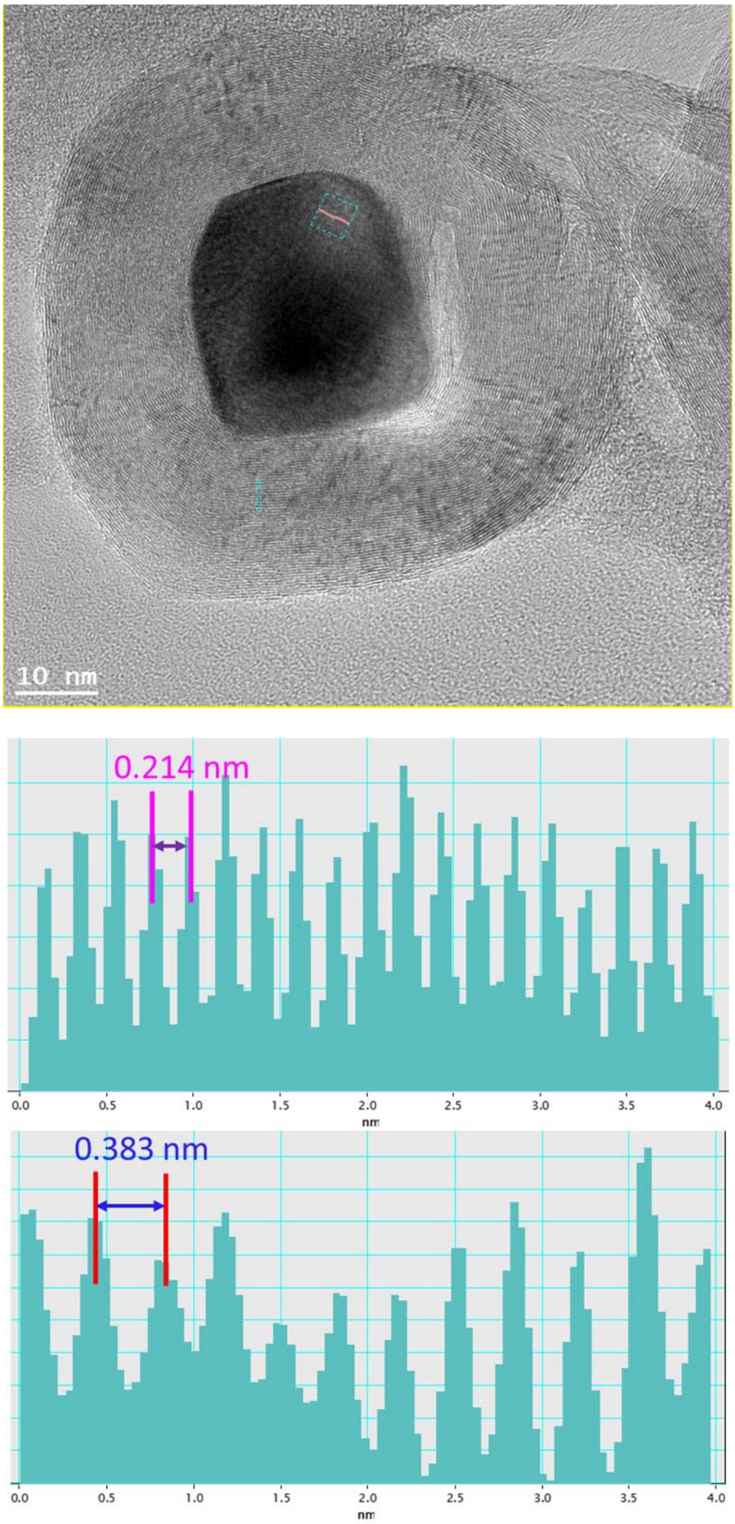

Supplementary Figure 26 | HRTEM image of the Co nanoparticles covered by a thin graphitic carbon shell of several nm in thickness. The lattice *d*-spacing of 0.214 nm (medium) and 0.383 nm (bottom) are corresponding to the $\{101\bar{1}0\}$ plane in hcp-Co and graphite, respectively.

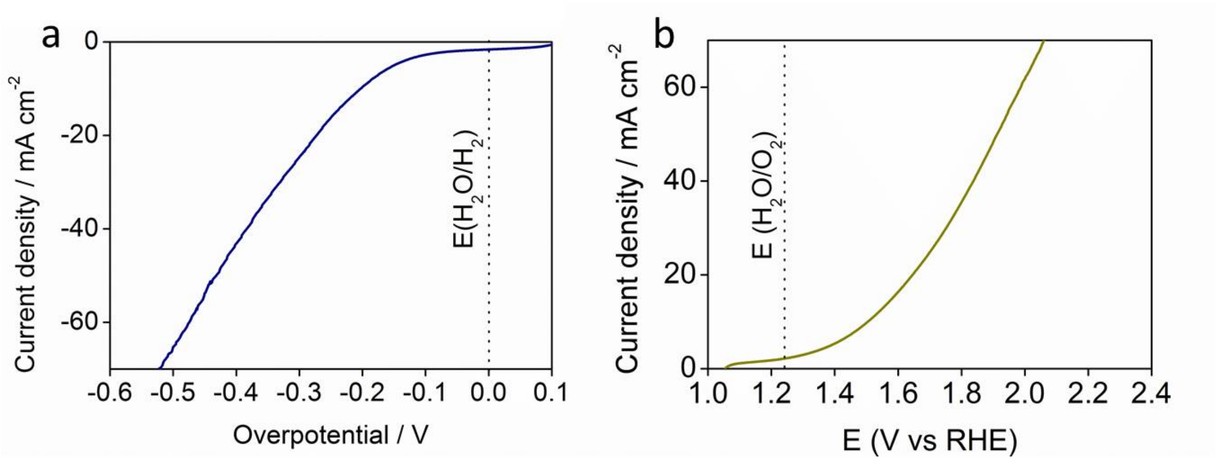

Supplementary Figure 27 | Without IR-corrected LSV curves of (a) HER and (b) OER for sample HNDCM-100,000-1000/Co, respectively.

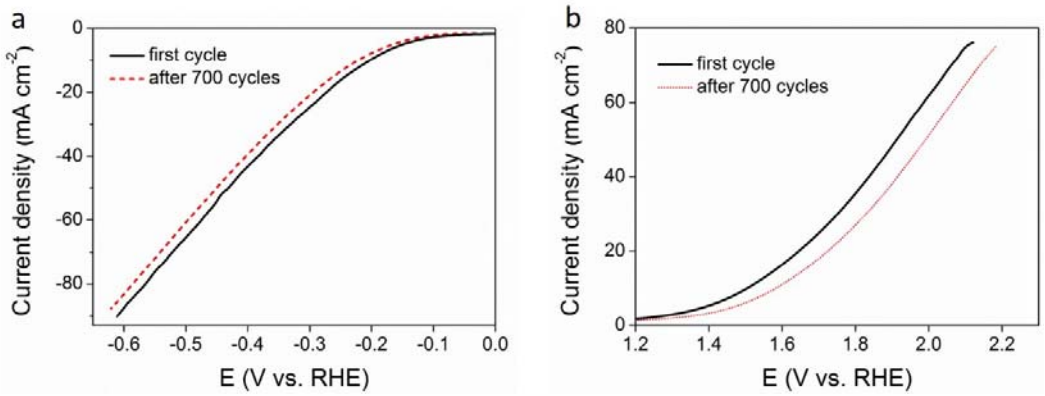

Supplementary Figure 28 | The CV stability curves of the 100,000-1000/Co electrode in HER (a) and OER (b), respectively, before and after running the CV test for 700 cycles.

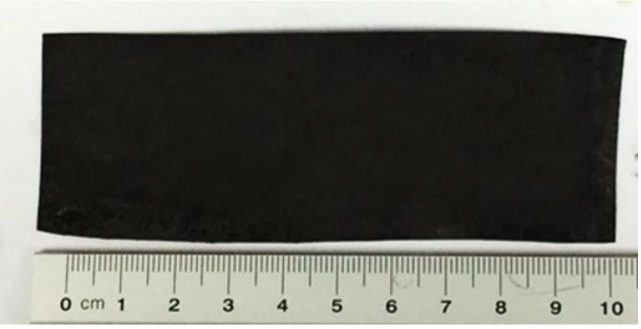

Supplementary Figure 29 | A large piece of HNDCM-100,000-1000/Co catalyst with a size of 10.5 x 3.5 cm^2 and thickness of ~70 µm. This membrane is the largest one that we can prepare using our carbonization oven at 1000 °C in our lab. The membrane size can be even larger if larger carbonization ovens are available.

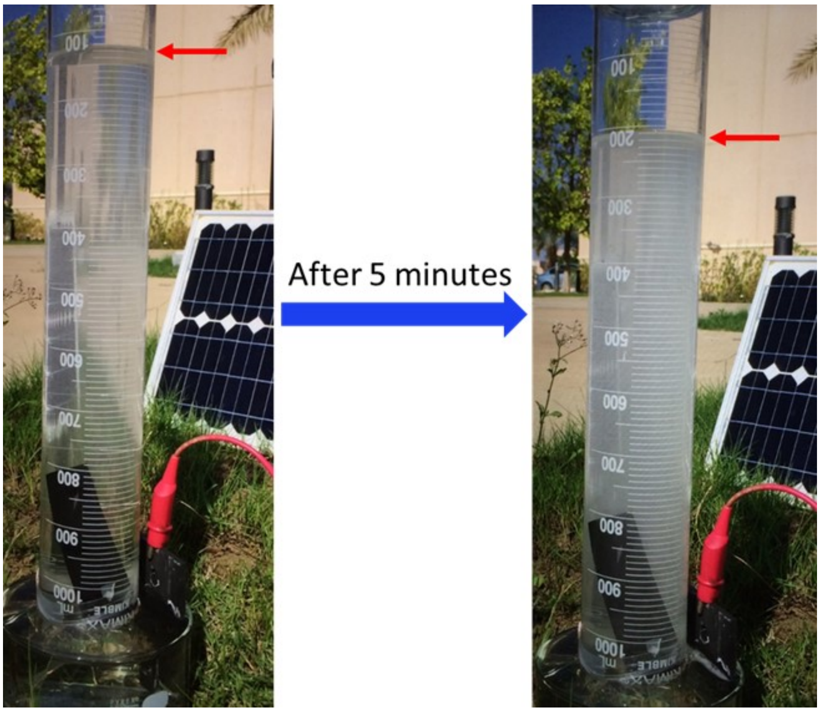

Supplementary Figure 30 | Photographs taken before and after HER driven by solar cell in 1 M KOH; 90 mL H_2 was collected within 5 minutes.The red arrows indicate the top line of the solution. (Note: this test was performed with unetched HNDCM-100,000-1000/Co film, which is even less efficient in H_2 production than the HCl-etched one.

Supplementary Table 1 | Degree of electrostatic complexation (DEC) of the GPPMs calculated by equations shown in Supplementary Fig. 23.

GPPM	S content determined by elemental analysis	DEC in average
GPPM-2000	12.6 %	11.7 %
GPPM-100,000	12.0 %	20.8 %
GPPM-250,000	12.1 %	19.4 %
GPPM-450,000	11.7 %	24.9 %
GPPM-3,000,000	11.8 %	23.6 %

Supplementary Table 2 | HER performance of HNDCM-100,000-1000/Co in this work, in comparison with several representative results with high performance non-noble metal based catalysts from recent publications.

Catalyst	Current density j (mA cm^{-2})	Overpotential (vs. RHE) at the corresponding j	Condition	*References*
MoB	10	225 mV	alkaline	*Angew. Chem., Int. Ed.* **51**, (12703-12706) 2012. (S4)
MoC	10	> 250 mV	alkaline	*Angew. Chem. Int. Ed.* 126, (6525 –6528), 2014. (S5)
**Co-NRCNT**	**10**	**370 mV**	**alkaline**	***Angew. Chem., Int. Ed.*, 53, (4372-) 2014. (S6)**
HNDCM-100,000-1000/Co	10	158 mV	alkaline	*This work*
CoOx@CN	10	232 mV	alkaline	*J. Am. Chem. Soc.* **137**, (2688−2694) 2015 (S7)
Nanoporous MoS_2	10	270 mV	acid	*Nature Mater.* **11**, (963-969) 2012. (S8)
Au supported MoS_2	0.2	150 mV	acid	*Science* **317**, (100-102) 2007. (S9)
Exfoliated WS_2/MoS_2 nanosheets	10	187-210 mV	acid	*Nature Mater.* **12**, (850-855) 2013. (S10); *J. Am. Chem. Soc.* **135**, (10274-10277) 2013. (S11)
MnNi	10	360 mV	Alkaline	*Adv. Funct. Mater.* **25**, (393-399) 2015. (S12)

Supplementary Table 3 | Data collected for Supplementary Fig. 18, the normalized results of different N contents

Samples	N1 %	N 2%	N 3%	N4 %	N 5%
HNDCM-100,000-800	32.1	13.0	35.3	9.4	10.2
HNDCM-100,000-900	18.5	10.4	43.6	15.3	12.2
HNDCM-100,000-1000	17.1	14.8	41.6	18.9	7.6

The fitted XPS peaks for N1s orbit of HNDCM-100,000-y (y=800, 900, and 1000) can be deconvoluted into five different bands at ~398.1, 399.5, 400.7, 402.1, and 404.6 eV, which correspond to pyridinic (N1), pyrrolic (N2), graphitic (N3), oxidized pyridinic (N4) and chemisorbed oxidized nitrogen (N5), respectively. These various N species lead to different chemical/electronic environments of neighboring carbon atoms and hence different electro-catalytic activities. The curve fitting and the corresponding normalized results indicate a conversion from pyridinic to graphitic nitrogen with increasing temperature, for example, the contents of pyridinic N in HNDCM-100,000-800, HNDCM-100,000-900 and HNDCM-100,000-1000 are 32.1%, 18.5% and 17.1%, respectively, which is consistent with previous reports on N-doped carbon materials[13].

Supplementary References:

1. Yang, S. *et al*. Efficient synthesis of heteroatom (N or S)-doped graphene based on ultrathin graphene oxide-porous silica sheets for oxygen reduction reactions. *Adv. Funct. Mater*. **22**, 3634-3640 (2012).

2. Zhang, D. *et al*. Electronic structure of graphite oxide and thermally reduced graphite oxide. *Carbon* 49 1362-1366 (2011).

3. Liang, C., Dai, S. & Guiochon, G. A graphitized-carbon monolithic column. *Anal. Chem.* **75**, 4904-4912 (2003).

4. Vrubel, H. & Hu, X. Molybdenum boride and carbide catalyze hydrogen evolution in both acidic and basic solutions. *Angew. Chem., Int. Ed.* **51**, (12703-12706) 2012.

5. Wan, C., Regmi, Y. N. & Leonard, Brian M. Multiple phases of molybdenum carbide as electrocatalysts for the hydrogen evolution reaction. *Angew. Chem., Int. Ed.* **126**, 6525-6528 (2014).

6. Zou, X. *et al*. Cobalt-embedded nitrogen-rich carbon nanotubes efficiently catalyze hydrogen evolution reaction at all pH values. *Angew. Chem., Int. Ed*. **53**, (4372-4375) 2014.

7. Jin, H. *et al*. In situ cobalt-cobalt oxide/N-doped carbon hybrids as superior bifunctional electrocatalysts for hydrogen and oxygen evolution. *J. Am. Chem. Soc*. **137**, 2688-2694 (2015).

8. Kibsgaard, J., Chen, Z., Reinecke, B. N. & Jaramillo, T. F. Engineering the surface structure of MoS_2 to preferentially expose active edge sites for electrocatalysis. *Nature Mater.* **11**, (963-969) 2012.

9. Jaramillo, T. F. *et al*. Identification of active edge sites for electrochemical H_2 evolution from MoS_2 nanocatalysts. *Science* **317**, (100-102) 2007.

10. Voiry, D. *et al*. Enhanced catalytic activity in strained chemically exfoliated WS_2 nanosheets for hydrogen evolution. *Nature Mater.* **12**, (850-855) 2013.

11. Lukowski, M. A. *et al*. Enhanced hydrogen evolution catalysis from chemically exfoliated metallic MoS_2 Nanosheets. *J. Am. Chem. Soc.* **135**, (10274-10277) 2013.

12. Ledendecker, M., Clavel, G. Antonietti, M. & Shalom, M. Highly porous materials as tunable electrocatalysts for the hydrogen and oxygen evolution reaction. *Adv. Funct. Mater.* **25**, (393-399) 2015.

13. Zhang, J. Zhao, Z., Xia, Z. & Dai, L. A metal-free bifunctional electrocatalyst for oxygen reduction and oxygen evolution reactions. *Nat. Nanotech.* **10**, 444-452 (2015).
